# Accurate spliced alignment of long RNA sequencing reads

**DOI:** 10.1093/bioinformatics/btab540

**Published:** 2021-07-24

**Authors:** Kristoffer Sahlin, Veli Mäkinen

**Affiliations:** Department of Mathematics, Science for Life Laboratory, Stockholm University, 106 91 Stockholm, Sweden; Department of Computer Science, University of Helsinki, 00014 Helsinki, Finland

## Abstract

**Motivation:**

Long-read RNA sequencing technologies are establishing themselves as the primary techniques to detect novel isoforms, and many such analyses are dependent on read alignments. However, the error rate and sequencing length of the reads create new challenges for accurately aligning them, particularly around small exons.

**Results:**

We present an alignment method uLTRA for long RNA sequencing reads based on a novel two-pass collinear chaining algorithm. We show that uLTRA produces higher accuracy over state-of-the-art aligners with substantially higher accuracy for small exons on simulated and synthetic data. On simulated data, uLTRA achieves an accuracy of about 60% for exons of length 10 nucleotides or smaller and close to 90% accuracy for exons of length between 11 and 20 nucleotides. On biological data where true read location is unknown, we show several examples where uLTRA aligns to known and novel isoforms containing small exons that are not detected with other aligners. While uLTRA obtains its accuracy using annotations, it can also be used as a wrapper around minimap2 to align reads outside annotated regions.

**Availabilityand implementation:**

uLTRA is available at https://github.com/ksahlin/ultra.

**Supplementary information:**

Supplementary data are available at *Bioinformatics* online.

## 1 Introduction

The transcriptome has been identified as an important link between DNA and phenotype and is therefore analyzed in various biological and biomedical studies. For these analyses, RNA sequencing has established itself as the primary experimental method. Some of the most common transcriptome analyses using RNA sequencing data include predicting and detecting isoforms and quantifying their abundance in the sample. These analyses are fundamentally underpinned by the alignment of reads to genomes. As a transcriptomic read can contain multiple exons, alignment algorithms are required to handle split alignment of a read to multiple exonic regions of the genome, referred to as a spliced alignment.

Spliced alignment is a challenging computational problem, and a plethora of different alignment algorithms have been proposed for splice alignment of short-read RNA-seq, with some of the key algorithmic advances given in TopHat (Trapnell *et al.*, 2009), STAR (Dobin *et al.*, 2013), HISAT (Kim *et al.*, 2015), GMAP (Wu *et al.*, 2016) and HISAT2 (Kim *et al.*, 2019). While short-read RNA sequencing has shown unprecedented insights into transcriptional complexities of various organisms, the read-length makes it difficult to detect isoforms with complicated splicing structure and limits quantification of isoform abundance (Zhang *et al.*, 2017).

Long-read transcriptome sequencing protocols such as Pacific Biosciences (PacBio) Iso-Seq sequencing (Wang *et al.*, 2016) and Oxford Nanopore Technologies (ONT) cDNA and direct RNA sequencing (Workman *et al.*, 2019) are now establishing themselves as the primary sequencing techniques to detect novel isoforms. Long-read sequencing technologies can sequence transcripts from end to end, providing the full isoform structure and therefore offer accurate isoform detection and quantification. Such protocols have opened up the possibility to investigate the isoform landscape for genes with multiple gene copies (Sahlin *et al.*, 2018) and complex splicing patterns (Tseng *et al.*, 2019), as well as to accurately decipher alleles (Tilgner *et al.*, 2014) and cell-specific (Gupta *et al.*, 2018) isoforms. However, the long-read technologies also offer new algorithmic challenges because of the higher error rate and longer sequencing length which makes most short-read alignment algorithms unsuitable for long-read splice alignment (Križanović *et al.*, 2018). Therefore, long transcriptomic reads have, similarly to short reads, prompted splice alignment algorithm development. Some short-read aligners have been modified for long-read splice alignment (Dobin *et al.*, 2013; Wu *et al.*, 2016), while other aligners have been designed for splice alignment of long reads (Boratyn *et al.*, 2019; Li, 2018; Liu et al., 2019; Marić *et al.*, 2019). A recent method also suggested improving long-read splice alignments using ensemble prediction of splice sites (Parker *et al.*, 2021). First, splice sites present in the sample are predicted using an ensemble of reads aligned in the region. In the second step, reads are aligned again using the predictions as a guide. There are also methods for post-correction of long-read splice alignments using ensemble-based predictions (Workman *et al.*, 2019). However, methods that use alignments from multiple reads to form consensus splice site predictions may over-correct less abundant splice sites and other rare events. Due to this limitation, it is desirable to have an accurate aligner that individually considers the best alignment for each read.

A particularly challenging task of long-read splice alignment is alignment to small exons (<30nt). Firstly, because of their length, small exons can be highly repetitive in the genome and be shorter than the required seed match length of the aligner. Secondly, even if the size is larger than the minimum seed match length, a small exon is less likely to contain seed matches if there are errors present. The inability to align a read to small exons may cause downstream analysis tools to predict and quantify erroneous isoforms. In addition, we show in this study that splice aligners that use junction-specific alignment penalties can create spurious junctions by overfitting alignments to canonical splice sites such as GT-AG junctions.

To alleviate these limitations, we have designed and implemented a splice alignment algorithm uLTRA that aligns long-reads to a genome using an exon annotation. uLTRA uses a novel two-pass collinear chaining algorithm. In the first pass, uLTRA, similarly to Li (2013), uses maximal exact matches (MEMs) between reads and the transcriptome as seeds. Due to their variable lengths, MEMs provide more information on the relevance of the hit than fixed-length seeds employed by many seed-and-extend methods (Dobin *et al.*, 2013; Kent, 2002; Li, 2018; Liu et al., 2019; Marić *et al.*, 2019; Wu *et al.*, 2016). Candidate genes regions are then identified from the MEM chaining solution. In the second pass, we design a novel chaining algorithm that aims to form a tiling of exon segments onto the read. This algorithm allows approximate sequence matches and incorporates approximate matches, overlap and gap costs into the formulation. The second pass also includes exons of the identified candidate gene region(s) without a MEM hit. This inclusion allows alignment to very small exons, which is the primary string of uLTRA, and differ from other two-pass alignment methods such as deSALT (Liu et al., 2019) and Graphmap2 (Marić et al., 2019). However, since uLTRA relies on annotations to perform alignments around annotated gene regions, the method is limited to finding isoforms in annotated regions. Therefore, to make uLTRA more broadly applicable, uLTRA also includes a setting where it wraps around minimap2 (default). In this setting, uLTRA uses minimap2’s primary alignments for reads aligned outside the regions indexed by uLTRA and chooses the best alignment of the two aligners for reads aligned in gene regions. This setting allows uLTRA to use minimap2 to detect novel transcripts in unannotated regions, and uLTRA’s accuracy in annotated gene regions.

We demonstrate using simulated datasets that uLTRA, both as a stand-alone aligner and as a wrapper around minimap2, produces much more accurate alignments than other aligners, particularly for small exons. We also use a dataset with ONT sequencing of synthetic SIRV transcripts (known isoforms) to demonstrate that uLTRA aligns more reads to transcripts that are known to be in the sample. Furthermore, we show on biological datasets from both PacBio and ONT that uLTRA aligns more reads to annotated isoforms and has alignments to more distinct isoform structures. Finally, we demonstrate that uLTRA produces alignments to known and novel isoform structures in the PacBio Alzheimer dataset that are not found by other aligners. These isoform structures come from genes that have been studied or linked to Alzheimer’s disease and motivate the utility of our method for a range of downstream analysis tasks such as isoform prediction and detection, splice-site analysis, isoform quantification and more. uLTRA is available at https://github.com/ksahlin/ultra.

## 2 Materials and methods

### 2.1 uLTRA overview

**Fig. 1.**
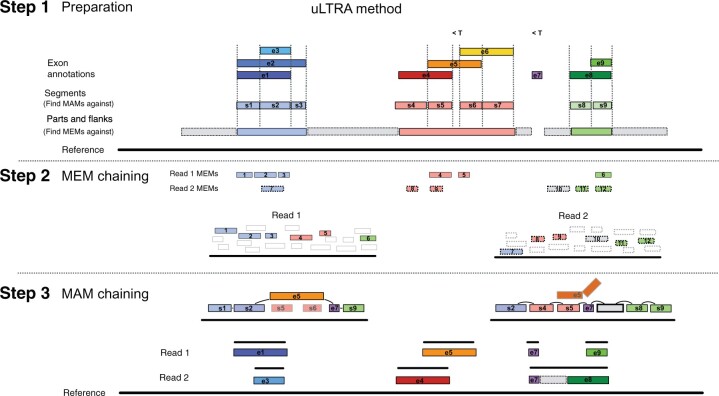
Overview of the uLTRA alignment algorithm. (Step 1) Segments (in color labeled sX, X∈[1,9]), parts (in color) and flanks (in grey) are stored and indexed for alignment. Small exons and segments below a threshold (indicated with <T in the figure) are not indexed for MAM chaining but stored for the MAM chaining. (Step 2) In the alignment step, MEMs in the reads to the parts and flanks are computed. Collinear chain(s) of MEMs covering as much of the read as possible are obtained for each read. Solutions may contain MEMs from intronic regions as is the case for read 2 in the figure (MEM number 10). The solution in step 2 consists of MEMs that overlap segments and/or flanks that are linked to gene IDs. (Step 3) All the segments and flanks assigned to the same gene IDs as the MEMs in the solution of step 2, including small exons and segments excluded from the indexing, are retrieved and aligned to the read to form a set of MAMs. Collinear chains of MAMs are found by optimizing for coverage and alignment identity according to the dynamic programming formulation in Section 2.4. The segments that are part of the solution to the MAM chaining are illustrated with solid black border in the figure, while the segments that are not part of the solution appear in gray. Some segments and exons might not align well with the read in this step, as is the case with e5 to read 2, and are illustrated by the broken alignment. The collinear chaining solution of MAMs is used to produce the final alignment of the read to the genome

uLTRA solves the algorithmic problem of chaining with overlaps to find alignments. The method consists of three steps. An overview of uLTRA is shown in Figure 1. We first construct subsequences of the genome referred to as parts, flanks and segments (Fig. 1A; details in Section 2.2). This step corresponds to the indexing step commonly performed by aligners, where the data structures do not need to be reconstructed for new sequencing datasets of the same organism. Our indexing strategy is unique and tailored to the design of the algorithm.

To align reads, uLTRA first finds maximal exact matches (MEMs) between the reads and the parts and flanks using slaMEM (Fernandes and Freitas, 2014) (Fig. 1B). Each read will have a set of MEMs to the genome reference sequences (e.g. a set of chromosomes). Furthermore, we partition the instances within chromosomes if two consecutive MEMs on the chromosome are separated by more than a parameter threshold provided to uLTRA. For each instance, uLTRA finds a collinear chain of MEMs covering as much of the read as possible (allowing overlaps of MEMs in the read). We use Algorithm 1 in Mäkinen and Sahlin (2020) to find such optimal chaining (see Step 2 in Section 2). The optimal solutions to the instances produce candidate alignment sites.

In the third step, we design a dynamic programming formulation that aims to chain together the combination of genomic segments that best fits the read. Each solution to the MEM chaining is processed as follows. The MEMs in the chaining solution overlap distinct segments on the genome (segments defined in Section 2; see Fig. 1B for illustration). Each segment belongs to a set of at least one gene. uLTRA aligns these segments together with all small exons (from the same genes) using edlib (Šošić and Šikić, 2017). Each such alignment produces a maximal approximate match (MAMs; defined in Section 2), and uLTRA uses all MAMs with alignment accuracy greater than a threshold T as input for the next chaining problem. There can be several MAMs of the same segment (or small exon) within a read. In the chaining of MAMs, we roughly optimize the total weight of MAMs covering the read while penalizing gaps and overlaps between MAMs. Here, weight is defined by the alignment accuracy and the length of the match (see Step 3 in Section 2). The final set of MAMs produced from the optimal solution(s) constitutes a final set of segments on the genome (Fig. 1C). Finally, we align the final set of segments to the read using parasail (Daily, 2016) (semi-global mode), which produces the final alignment(s) and cigar strings to the genome.

When uLTRA is used as a wrapper around minimap2, it runs minimap2 and parses minimap2’s alignments to find primary alignments outside the regions indexed by uLTRA. These alignments are not considered for alignment with uLTRA. uLTRA then proceeds to align all remaining reads. In a final step, uLTRA compares the reads that have been aligned with both aligners and selects the best alignment based on edit distance to the genome. The final output SAM-file consists of the best alignments to uLTRA-indexed regions and the alignments of minimap2 outside the regions indexed by uLTRA.

### 2.2 Step 1: indexing

A part is defined as the smallest genomic region fully covering a set of overlapping exons (Fig. 1). By construction, parts are disjoint regions of the genome. Flanks are constructed by taking regions of size F nucleotides downstream and upstream of parts. If two parts are separated with a distance of less than F nucleotides, then the non-overlapping region between the two parts is chosen as a flank region (Fig. 1). By construction, flanks are disjoint regions, both in relation to each other and to parts. Finally, segments are constructed from start and end coordinates of exons. Segments are constructed for each part individually as follows. For a sorted array of exon start and stop coordinates within a part, a segment is constructed for each pair $\left(x_{i},x_{i+1}\right)$ of adjacent coordinates in the array if $x_{i+1}-x_{i}\geq X$ where *X* is a parameter to uLTRA (set to 25). If $x_{i+1}-x_{i}<X$, uLTRA iteratively attempts to add segments in each direction until success. That is, uLTRA attempts to add $\left(x_{i-k},x_{i+1}\right)$ and $\left(x_{i},x_{i+1+k}\right)$ for k=1,2,…, until the first success in each direction. Finally, there may be parts where y−x<X (see exon e7 in Fig. 1). Small segments, exons or parts have a lower probability of containing a MEM, and may therefore not have a MAM. We address this complication as follows. uLTRA stores all exons and segments smaller than a threshold in a container that links gene ID to the small segments. This data structure will be queried, and all small segments will be included, whenever there are MEMs to segments linked to the same gene ID.

### 2.3 Collinear chaining with MEMs

A Maximal Exact Match (MEM) ([a..b],[c..d]) means that a genome segment [a..b] matches a read segment [c..d], and that such a match cannot be extended in either direction. We use notation A[i].x to denote the endpoints of MEMs for x∈a,b,c,d. Let array A[1..n] contain the MEMs. A chain *S* is a collinear subset of *A*, meaning that S[i].a<S[i+1].a and S[i].c<S[i+1].c for 0<i<n [i.e. satisfying the weak precedence (Mäkinen and Sahlin, 2020)]. *Coverage*(*S*) is defined as the number of identities in an alignment induced by *S*, i.e. the length of the anchor-restricted LCS (longest common subsequence) of reference and the read, where anchor now means a MEM (Mäkinen and Sahlin, 2020): If there are no overlaps between MEMs in chain *S*, *Coverage*(*S*) is the overall length of MEMs in *S*, but if there are, the score is adjusted by adding only the minimum length of the non-overlapping parts of the consecutive MEM intervals (Mäkinen and Sahlin, 2020). Here, we look for chains that have no overlaps in the genome, so for finding *S* that maximizes *Coverage*(*S*), we use Algorithm 1 in Mäkinen and Sahlin (2020) that runs in O(n log n) time.

### 2.4 Collinear chaining with MAMs

We refer to an approximate match, as an alignment of a genome segment [a..b] to a read segment [c..d] with an accuracy higher than a threshold (parameter to uLTRA). Here, accuracy is defined as the number of matches divided by the length of the alignment. We find approximate matches of the genome segment by aligning it in semi-global mode to the read using edlib (Šošić and Šikić, 2017). The length of the alignment is defined by the genome segment’s first and last nucleotide coordinates. A Maximal Approximate Match (MAM) ([a..b],[c..d]) means that genome segment [a..b] matches approximately read segment [c..d] and that no other approximate match has higher accuracy on the read. Intuitively, a MAM can be seen as the semi-global alignment that has the highest accuracy where the segment is forced to be fully aligned. Furthermore, we let λ∈[0,1] be the penalty for each nucleotide that overlaps (on the read) between two MAMs and δ∈[0,1] the penalty for the distance between two MAMs (on the read). Let array A[1,…,N] contain the MAMs where we use the following notation: A[i].a,A[i].b,A[i].c,A[i].dA[i].acc to denote the genome start, genome stop, read start, read stop and accuracy of MAM *i*. Let S[1,…,m] be a chain of the MAMs in *A* under the weak precedence constraint (Mäkinen and Sahlin, 2020). For two MAMs *x*, *y* in *A*, we introduce the following functions. Let v(x)=(x.d−x.c), o(x,y)=max{0,x.d−y.c} (the overlap), and d(x,y)=0,y.c−x.d (the distance between MAMs) on the read, then the *score*(*S*) of a MAM-chain is defined as

$$\begin{array}{c} \mathrm{score}(S)=\sum_{i=1}^{m}(v(S[i])-o(S[i-1],S[i]))S[i].\mathrm{acc}\\ \;-\lambda o(S[i-1],S[i])-\delta d(S[i-1],S[i]), \end{array}$$

where o(S[0],S[1])=d(S[0],S[1])=0

We find the chain $S^{\max}=\max_{\text{chains}S}s\mathrm{core}(S)$. This formulation intuitively selects the solution with the best coverage and accuracy, while penalizing overlapping MAMs or MAMs that occur far apart. This formulation is solved with a dynamic programming algorithm: Sort array A[1,…,N] by values A[i].a. Let W[0,…,N] be the target array, where we wish to store for each W[i] the maximum score over chains ending at MAM A[i]. To compute W[i], one can consider adding A[i] to chains ending at A[i′],i′<i with A[i′].c<A[i].c. This increases the score by w(i′,i)=(v(A[i])−o(A[i′],A[i]))A[i].acc−λo(A[i′],A[i])−δd(A[i′],A[i]). After initializing W[0]=0, we can set W[i] to the maximum over W[i′]+w(i′,i) for 0≤i′<i with A[i′].c<A[i].c from left to right, and the maximum scoring chain can be traced back starting from the maximum value in W[1,..,N]. Although this computation takes quadratic time, in practice the instances of segments are small enough to be solved quickly. It is not known whether our formulation allowing weighted hits, overlap and gap penalties can be solved in subquadratic time, although recent breakthroughs have been made for chaining problems allowing overlap and gap costs (Jain *et al.*, 2021).

### 2.5 Wrapping around minimap2

uLTRA can be used as a wrapper around minimap2 to detect alignments outside annotated regions. In this mode, uLTRA first runs minimap2. After reads have been aligned with minimap2, uLTRA parses minimap2’s alignments to find reads with primary alignments outside the regions indexed by uLTRA. A read with more than a fraction of *X* nucleotides (parameter to uLTRA; *X *=* *0.1 used here) out of the total aligned nucleotides is considered genomic and not realigned with uLTRA. We use an interval-tree data structure to hold the indexed regions to find overlap of a read and indexed regions. This permits an O(logQ) query time, where *Q* is the number of intervals on the chromosome to which the read is aligned. The alignments that are classified as genomic are not aligned with uLTRA. uLTRA then proceeds to align all remaining reads. Instead, uLTRA will report minimap2’s primary alignments for these reads. uLTRA then proceeds to align all reads not classified as genomic as described. In a final step, uLTRA compares the reads that have been aligned with both aligners and selects the best alignment based on edit distance to the genome. The final output SAM-file consists of the best alignments to uLTRA-indexed regions and minimap2’s alignments of genomic reads. As most genomes have incomplete exon annotations, the minimap2 wrapping-mode is the default setting to uLTRA. In cases where the annotation is guaranteed to be complete, such as for smaller organisms or controlled SIRV datasets, uLTRA can be run as a stand-alone tool.

### 2.6 Implementation

#### 2.6.1 Chaining of MEMs

In the implementation, the optimal solution instance is found through backtracking. If several possible traceback paths lead to the same optimal value for a given optimal value in the traceback vector, uLTRA will always choose the closest MEM, i.e. the one with the highest index *j*. This means that the MEM with the closest genomic coordinate is chosen if several exist.

If several optimal chaining solutions are found, i.e. several positions in the vector traceback vector have the optimal value, uLTRA will report all of the solutions by backtracking each instance (as described above). This is not a rare case since there can be identical or highly similar gene copies annotated on the genome that give the same optimal value.

Since each read can have several chaining instances to solve, uLTRA pre-calculates the theoretical maximum MEM coverage that an instance can have, which is upper bounded by the sum of all the regions covered by MEMs in the reads. uLTRA then solves the chaining instances by highest upper bound on coverage. If at any point the upper bound drops below a drop-off threshold (parameter to uLTRA) the current best solution uLTRA skips to calculate the rest of the instances. There is also a parameter to limit the number of reported alignments.

#### 2.6.2 Chaining of MAMs

MAMs are formed by aligning segments and exons with at least an alignment identity of X% (default 60), and in case of exons between 5 and 8 nucleotides in length, an exact match is required. Exons of 4 bp or less are ignored because of the potential blowup in the number of matches across the read. Similarly to the MEM chaining, the traceback will choose the MAM with the highest index *j*.

#### 2.6.3 Alignment reporting

The exons that are included in an optimal solution of the MAM chaining are concatenated into an augmented reference, and the read is aligned to this reference using parasail (Daily, 2016) in semi-global mode. The alignment score and cigar string are computed from the alignment. Among all MAM instances for a read, the highest scoring one is selected as the primary alignment. If a read has multiple best scoring alignments, the one with the shortest genomic span of the alignment is reported, and if still a tie, an alignment matching the annotated splice sites is preferred.

A read is assigned as unaligned if the alignment score is lower than X·m·r, where *r* is the read length, *m* is the match score (set to 2 in parasail; see Supplementary Note SA for details) and *X* is a parameter to uLTRA (set to 0.5). The default setting roughly corresponds to classifying a read as unaligned if it has more than 25% errors, or if a larger segment of the read is from a region that is not included in the indexing.

#### 2.6.4 Output

uLTRA outputs alignments in SAM-file format with genomic coordinates as annotated by the transcript database. In addition, uLTRA outputs a transcript annotation of the alignment following the definitions in Tardaguila *et al.* (2018) (described in results) in the SAM-file in the optional field ‘CN’.

## 3 Results

### 3.1 Evaluation overview

We evaluated uLTRA against the two state-of-the-art transcriptomic long-read aligners minimap2 and deSALT. We also attempted to evaluate GraphMap2 but were unsuccessful in using the tool (see Supplementary Note SA). Several additional alignment methods can perform splice alignment of long transcriptomic reads such as BBMAP (Bushnell, 2014), GMAP (Wu *et al.*, 2016) STAR (Dobin *et al.*, 2013) and HISAT2 (Kim *et al.*, 2019). A recent benchmarking (Križanović *et al.*, 2018) showed that GMAP performed the best among the tools compared on long noisy reads from complex genomes such as the human genome. Additional recent methods not included in Križanović *et al.*, (2018) include Graphmap2 (Marić et al., 2019), minimap2 (Li, 2018), deSALT (Liu et al., 2019) and Magic-BLAST (Boratyn *et al.*, 2019). However, in (Liu et al. 2019), the authors showed that deSALT, minimap2 outperformed GMAP across a large range of datasets, while in Boratyn *et al.* (2019), which compared performance on both short and long reads, minimap2 performed the best for long noisy reads. Therefore, we compare uLTRA to the more recent and best performing aligners minimap2 and deSALT. We run minimap2 and deSALT both with and without annotations as the two aligners support such modes. A tool that is run with annotations has ‘_GTF’ appended to its name. We used parameters for minimap2 and deSALT for the Iso-Seq and ONT datasets as recommended by the developers (Li, 2018). We also ran uLTRA both as a stand-alone tool (labeled uLTRA) and as a wrapper around minimap2 (labeled uLTRA_mm2). Details for how the aligners were run are found in Supplementary Note SA.

We used three simulated, one synthetic and two biological datasets from both ONT and PacBio Iso-Seq (Table 1) to evaluate the alignment algorithms. We used simulated datasets with known annotations to investigate the accuracy of spliced alignments as a whole, and of individual exons as a function of exon size. We used the synthetic SIRV data to investigate how aligners perform when aligning real sequencing reads to isoform structures known to be in the sample. Finally, for the biological data where we do not have the ground truth annotations we measured the concordance in alignments between alignment methods. We also demonstrate that relying on alignment concordance as a proxy for alignment accuracy can be misleading due to similar alignment biases between aligners. We also report runtime and memory usage.

**Table 1.** Datasets included in evaluation SIRV genome

Technology	Dataset	Nr reads	Median read length	Median error rate	Genome	Annotation
Simulated	ENS	234 207	890	0.0%	GRCh38.p12	Gencode v34^b^
Simulated	SIM_ANN	1 000 000	864	8.6%	GRCh38.p12	Gencode v34^b^
Simulated	SIM_NIC	1 000 000	1272	8.6%	GRCh38.p12	Gencode v34^b^
ONT	SIRV	1 514 274	538	6.9%	SIRV annotation C_170612a	
ONT	DROS	3 646 342	559	7.0%^a^	BDGP6.28	Ensembl v100
Iso-Seq	ALZ	4 277 293	2699	1.2%^a^	GRCh38.p12	Gencode v34^b^

a Measured from minimap2’s alignments. Due to biological sequence variations, the error rate may be lower than the number presented here.

b Includes alternative haplotypes.

### 3.2 Alignment accuracy

We used three in silico datasets to test the alignment accuracy in a controlled setting (Table 1). First, we used 234 207 distinct cDNA sequences downloaded from ENSEMBL (denoted ENS) without introducing any simulated errors. We then simulated a dataset of 1 000 000 reads uniformly at random from the 234 207 ENSEMBL sequences with a mean error rate of 8.6% (denoted SIM_ANN for simulated annotated transcripts). Finally, to test the ability to align to transcripts containing novel combinations of exons, we simulated a dataset with the same error rate as SIM_ANN, which we call SIM_NIC for simulated Novel-In-Catalog transcripts. This dataset consists of reads from transcripts with novel exon combinations that we generated from gencode annotations (release 34, including haplotype scaffold annotations). See Supplementary Note SB for details on the simulations. Since we have the true exon annotation of each read, we classify the read alignments as correct, inexact, exon difference, incorrect location and unaligned. In order for an alignment to be classified as correct, no splice site in the alignment can have an offset to the true splice site with more than 15 nucleotides, which is more than the largest indel error we observed in our simulated data, indicating an alignment error rather than a sequencing error. For details of these classifications, see Supplementary Note SB.

**Fig. 2.**
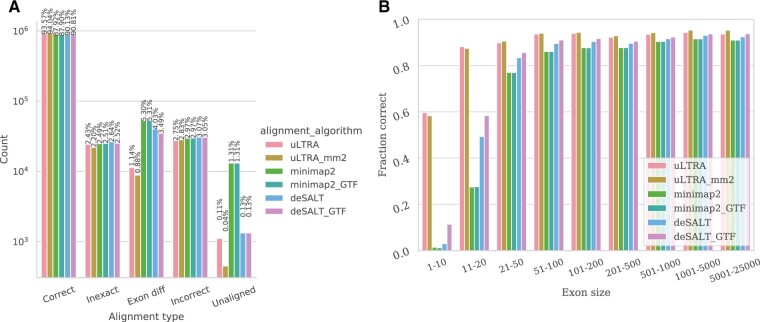
Alignment results on simulated data for the SIM_ANN dataset. (**A**) Percentage of reads in each respective category. (**B**) The fraction of correctly aligned exons (*y*-axis) as a function of exon size (*x*-axis)

For SIM_ANN, which contains simulated reads from annotated transcripts, uLTRA and uLTRA_mm2 have the highest fraction of correct alignments (93.6% and 94.0%) with a 3.2 percentage point increase compared to the second-best performing tool deSALT_GTF (Fig. 2A). uLTRA and uLTRA_mm2 also substantially reduce errors classified as exon differences compared to the other aligners (Fig. 2A). Furthermore, we observed that both uLTRA and uLTRA_mm2 achieve considerably higher accuracy than other aligners for small exons (Fig. 2B). For comparison, when minimap2 is run as a stand-alone tool, it has an accuracy of 87.9% (Fig. 2A), which indicated that the majority of uLTRA’s alignments are preferred. We observed similar trends for the ENS dataset (Supplementary Fig. S1).

As for the SIM_NIC, which contains only reads with novel combinations of exons, uLTRA’s and uLTRA_mm2’s accuracy is as high as for the ENS and SIM_ANN datasets (Supplementary Fig. S2A). However, on this dataset uLTRA has a 9.6 percentage point more correctly aligned reads compared to the second-best performing aligner deSALT_GTF (Supplementary Fig. S2A), and a 24.4 percentage point increase to minimap2. Our results show that the accuracy is substantially lower for the other aligners across exons sizes on this dataset (Supplementary Fig. S2B). The differences with SIM_NIC compared to the other two simulated datasets is that the reads (i) can be simulated from alternative haplotype sequences and novel isoform structures, and (ii) that the datasets contain, on average, longer reads than the ENSEMBL annotation (Table 1).

We also compared results in a stringent setting when we did not allow any offset between the true and aligned splice sites (Supplementary Fig. S3). In this setting, uLTRA and uLTRA_mm2 have a more substantial accuracy improvement relative to the other aligners. While uLTRA_mm2 only has a 3.7 percentage point increase compared to the second-best performing tool deSALT_GTF on the ENS dataset (Supplementary Fig. S3A), this gap significantly increases when errors are present to 15.5 and 47.2 between uLTRA_mm2 and deSALT_GTF for the SIM_ANN (Supplementary Fig. S3B) and SIM_NIC (Supplementary Fig. S3C), respectively.

### 3.3 Splice site annotation performance on SIRV

While simulated data is good for comparisons due to the availability of ground truth annotations, it does not fully capture the error profiles present in sequencing data. We used a subset of 59 isoforms with distinct splice site positions from the ONT cDNA SIRV dataset (Sahlin and Medvedev, 2021) to investigate alignment performance around splice sites (for details see Supplementary Note SC). In this dataset we have a complete isoform annotation and the sequenced isoforms are known. We observed that uLTRA was able to align more reads to the isoforms, particularly to one isoform that contains an 8 nt long exon. deSALT and minimap2 did not align the large majority of reads that contained the exon (Supplementary Fig. S4E). Overall, uLTRA’s alignments were more equally distributed across the 59 isoforms, as is expected in the SIRV E0 mix (see Supplementary Note SC). More details about the analysis and results are described in Supplementary Note SC.

### 3.4 Biological data

We also used an Alzheimer brain Iso-Seq dataset (denoted ALZ) and an ONT cDNA sequencing dataset from Drosophila (Sahlin and Medvedev, 2021) (denoted DROS). Both datasets have been processed with respective bioinformatics pipelines to select only the reads containing full-length transcripts (for details see Supplementary Note SD).

We neither have the correct read annotations, nor are we guaranteed to have a complete gene annotation for the biological datasets, which presents a challenge when evaluating accuracy. We took the following approaches. We first compared the alignment algorithms according to the alignment categories defined in Tardaguila *et al.* (2018) (presented in the next section). Secondly, we looked at the alignment concordance between methods. Here, we investigated concordance with respect to both alignment location on the genome and concordance based on the alignments around exons. Thirdly, we provide several examples of uniquely detected isoforms by uLTRA (and uLTRA_mm2), which demonstrate the caveats with alignment concordance analysis without ground truth.

#### 3.4.1 Alignment categories on biological data

We classified alignments using the categories defined in Tardaguila *et al.* (2018). As in Tardaguila *et al.* (2018), we classify an alignment of a read to the genome as a Full Splice Match (FSM), Incomplete Splice Match (ISM), Novel In Catalog (NIC), Novel Not in Catalog (NNC) or NO_SPLICE. An FSM alignment means that the combination of splice junctions in the read alignment has been observed and annotated as an isoform. An ISM alignment means that the combination of splice junctions is in the annotation, but it is missing junctions compared to the annotated models in either the 3′ or 5′ end. A NIC alignment consists of junctions that all appear in the annotation, but not together in a single isoform. An NNC alignment means that the read aligns with at least one junction that is not in the annotation, while NO_SPLICE are all alignments without splice sites. These alignment categories are important for various downstream isoform detection methods such as SQANTI (Tardaguila *et al.*, 2018), TAMA (Kuo *et al.*, 2020) or TALON (Wyman *et al.*, 2019). See Tardaguila *et al.* (2018) for details regarding these definitions.

**Fig. 3.**
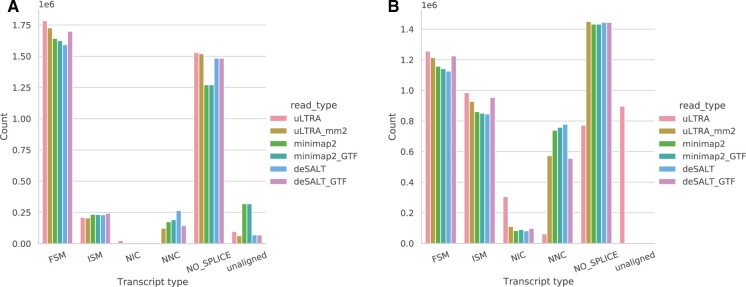
Number of reads annotated in different splicing categories for DROS (**A**) and ALZ (**B**)

Overall, the aligners and their different modes produce a similar distribution of the different alignment categories on both the DROS (Fig. 3A) and ALZ (Fig. 3B) datasets. We observe that uLTRA, uLTRA_mm2 and deSALT_GTF align more FSM reads than deSALT, minimap2 and minimap2_GTF. In the ALZ dataset, uLTRA has many unaligned reads due to a large fraction of reads (17.6%) aligning outside uLTRA indexed regions. This highlights the benefit of not being limited to alignments around gene regions when aligning transcriptomic data even for well annotated genomes. We observe that the other aligners, including uLTRA_mm2, have no unaligned reads. Instead, they attribute a larger fraction of reads in the category NO_SPLICE (Fig. 3B). It is known that a substantial fraction of reads in long-read transcriptome sequencing data is coming from so-called intra-priming reads (Tardaguila *et al.*, 2018). These reads are characterized by aligning without splice junctions to an unannotated genome location that contains a poly-A stretch downstream from their 3′ end. While not fully characterized, these reads are likely to be artifacts in the sequencing protocol and often filtered out in downstream analysis (Tardaguila *et al.*, 2018).

We further investigated concordance in alignments within the different categories between uLTRA_mm2, deSALT_GTF and minimap2. They represent the best setting for each aligner, respectively, based on our accuracy evaluation on simulated data (Fig. 2, Supplementary Figs S1 and S2) and alignment consistency analysis on the synthetic SIRV data (Supplementary Fig. S4).

#### 3.4.2 Alignment concordance on biological data

We looked at alignment concordance both with respect to genomic region (globally) and around exons (locally). A detailed description is found in Supplementary Note SE. Overall, we observed that 90.3% and 98.6% of all aligned reads had globally concordant alignments in DROS and ALZ, respectively (Supplementary Fig. S5). This indicates that the mapping region is largely consistent between aligners and that most of the variability occurs in alignments around exons. We also report local alignment concordance for each category, which was lower across each category (Supplementary Figs S6 and S7).

We also looked in more depth at the concordance of unique isoforms detected in the data that had FSM predictions (Supplementary Fig. S8). In total, 93.6% and 90.1% of the total unique isoforms with FSM alignments were aligned to by all the three methods on both datasets, for DROS and ALZ, respectively. Notably, we observe that uLTRA_mm2 had the most predicted isoforms which were also predicted by one of the other two aligners. Specifically, 2.2% (DROS) and 4.3% (ALZ) of the isoforms was predicted by both uLTRA_mm2 and deSALT_GTF, but not by minimap2. Similarly, 1.6% (DROS) and 1.9% (ALZ) of the isoforms was predicted by both uLTRA_mm2 and minimap2, but not by deSALT_GTF. This was substantially more than the shared predictions between deSALT_GTF and minimap2 that uLTRA_mm2 did not align to, which was only 0.1% and 0.4%, for DROS and ALZ, respectively. While we have no ground truth, the concordance may indicate that the robust performance that we observed for uLTRA_mm2 on simulated and synthetic datasets translates to biological datasets. However, we next looked at some isoforms that were uniquely detected by uLTRA_mm2, which highlight the limitations with concordance analysis without ground truth.

#### 3.4.3 uLTRA aligns to small exons not detected by other aligners

We further investigated some of the isoform structures uniquely aligned to by uLTRA_mm2 and observed that in several cases, uLTRA’s alignments were correct. We detected several instances in the ALZ dataset where uLTRA_mm2 aligned to both known and novel isoforms containing small exons that were not detected by other aligners (Supplementary Figs S10–S14). Several of these uniquely detected isoforms had high read support and came from genes such as AP2, APBB, HNRNPM, DCTN2, PRNP, MICU1, SEPTIN7 and APBB1, that have been studied in relation, or linked directly, to Alzheimer’s disease (Bagyinszky *et al.*, 2019; Calvo-Rodriguez *et al.*, 2020; Geuens *et al.*, 2016; Tanahashi and Tabira, 1999; Tian *et al.*, 2013; Wang *et al.*, 2018) or other neurodegenerative disorders (Boland *et al.*, 2018; Charbonnier *et al.*, 1997). For a detailed description of the results and analysis, see Supplementary Note SF.

In addition, we hypothesized that potential erroneous alignments by uLTRA_mm2 were present in the small fraction of unique isoforms with FSM predictions predicted by both deSALT_GTF and minimap2 that uLTRA did not align to (0.1% in DROS and 0.4% in ALZ, Supplementary Fig. S8). We investigated all the eight cases in the ALZ dataset that (i) has a read coverage over ten FSM reads and (ii) where minimap2 and deSALT agreed on a unique FSM isoform but uLTRA_mm2 did not. Strikingly, we found that in all cases but one, uLTRA_mm2 had better alignments than minimap2 and deSALT_GTF (Supplementary Figs S15–S17). However, we found a case where uLTRA_mm2 clearly failed to produce an alignment over a long intron (302 254nt on chr5, Supplementary Fig. S17). Our manual inspection showed three types of sources of errors in long-read splice alignment: (i) small exon misalignment (Supplementary Fig. S15), (ii) over-fitting canonical splice site (Supplementary Fig. S16) and (iii) alignment over long introns (Supplementary Fig. S17). Our analysis highlights that alignment concordance between aligners may not indicate correct alignment as concordance can come from the same algorithmic decisions between aligners such as customized alignment penalties for canonical regions or inability to align to very short exons.

### 3.5 Runtime and memory usage

We used a 128 Gb memory node with 20 cores and tested the tools using both 4 and 19 cores (leaving one core for the main process). We measured user time (total time from start to finish) and peak memory usage (highest memory usage across the program lifetime). Runtimes using 4 cores are shown in Table 2 and 19 cores in Supplementary Table S1. In general, we observe that deSALT and minimap2 is faster than uLTRA but the relative runtime performance decreases with the size of the organism and the length of the reads. For example, on the two largest datasets, SIM_NIC and ALZ, deSALT is about 2 times and 1.6 times faster than uLTRA. A computational bottleneck in uLTRA is the MEM finding using slaMEM. slaMEM trades speed for memory footprint. On the DROS and ALZ datasets, the MEM-finding step is accountable for 35% and 22% of the total uLTRA runtime when using 4 cores, and for over 60% when using 19 cores. As for the memory, minimap2 has the smallest peak memory footprint on most datasets (Supplementary Tables S2 and S3). However, overall, the tools use a comparable amount of memory. uLTRA_mm2 use more memory with more cores. For example, on the dataset with the largest peak memory footprint in our experiments (ALZ), uLTRA_mm2 has a peak memory usage of 64 Gb using 4 cores and 102 Gb using 19 cores. uLTRA in standalone mode uses slightly less memory than uLTRA. See Supplementary Note SG for a detailed discussion of the runtime and memory usage.

**Table 2.** Runtime of alignment using four cores

Dataset	uLTRA	uLTRA_mm2	minimap2	minimap2_GTF	deSALT	deSALT_GTF
ENS	52 min	1 h 11 min	43 min	45 min	18 min	**17 min**
SIM_ANN	2h 47 min	4h 00 min	2 h 42 min	2 h 48 min	**1 h 20 min**	1 h 23 min
SIM_NIC	3 h 21 min	6h 40 min	4 h 35 min	4 h 42 min	**1 h 46 min**	1 h 55 min
SIRV	35 min	50 min	13 min	13 min	**6 min**	7 min
ALZ	16 h 9 min	17h 32 min	**9 h 4 min**	9 h 47 min	10 h 16 min	10h 17 min
DROS	1 h 17 min	1h 37 min	**18 min**	25 min	23 min	23 min

## *Note*: Boldfaced values indicate shortest runtime.

 

## 4 Discussion

Splice alignment is an algorithmic problem central for the detection and prediction and quantification of isoforms. We have presented a novel splice alignment algorithm, and its implementation uLTRA. uLTRA aligns long transcriptomic reads to a genome using an annotation of coding regions. In addition, uLTRA can also run as a wrapper around minimap2. In this mode, it refines alignments around gene regions. uLTRA outputs alignments in SAM-format, and classifies the splice alignments according to the classification given in Tardaguila *et al.* (2018) under an optional tag in the SAM-file. We evaluated uLTRA on simulated, synthetic and biological data, and our analysis highlights some of the challenges with splice alignment and the current state-of-the-art approaches.

Using simulated data, we demonstrated uLTRA’s increased accuracy over other aligners. Particularly, uLTRA outperformed other state-of-the-art splice aligners when aligning reads to small exons. We also observed that uLTRA had high accuracy on the SIM_NIC dataset while the accuracy of other methods substantially decreased. Furthermore, the accuracy was lower across exons sizes on this dataset (Supplementary Fig. S2B). The differences with SIM_NIC compared to the other two simulated datasets are that the reads (i) can be simulated from alternative haplotype sequences and novel isoform structures, and (ii) that the dataset contains longer reads than the ENSEMBL annotation (Table 1).

We used synthetic ONT data to investigate the performance of alignment algorithms when aligning reads to known splice sites. Our experiments demonstrate that uLTRA aligns a much higher percentage of reads to known isoforms in the data. This holds true when running uLTRA as a wrapper around minimap2, indicating that uLTRA’s alignments are preferred based on edit distance of the alignments. Furthermore, uLTRA’s FSM alignments are distributed across the 59 isoforms with distinct splice-sites without indication of alignment bias toward specific isoforms as other aligners have (Supplementary Fig. S4).

On biological data, we found that uLTRA_mm2 had the most predicted isoforms which were also predicted by one of the other aligners. While there is no ground truth for the biological datasets, the high concordance indicates that the high accuracy we observed for uLTRA_mm2 on simulated and synthetic datasets translates to biological datasets. We also demonstrated several examples where uLTRA aligns reads to the correct isoform structure while the other aligners do not. We showed several examples where isoforms containing small exons were misaligned (Supplementary Figs S10, S12 and S15), where employing junction-specific alignment penalties may lead to concordant but erroneous alignments around junctions (Supplementary Figs S11 and S16), and where alignment fails over large introns (Supplementary Fig. S17). We observed cases where homopolymer differences in reads may lead to subtle alignment differences causing alignment to novel junctions (Supplementary Fig. S13). In summary, the examples we provide on biological data demonstrate that using simple concordance analysis between aligners to measure accuracy can be misleading. Furthermore, the examples (Supplementary Figs S10–S14) came from genes that have been studied or linked to Alzheimer’s disease with many of them highly abundant. As several of these isoforms may not be detected with other alignment software, we demonstrated the utility of uLTRA and highlighted the significance of further development of splice alignment techniques.

We observed a large fraction of reads in the biological dataset that came from genomic regions in Homo Sapiens and Drosophila, which are two well-annotated genomes. The large majority of these reads were aligned as NO_SPLICE (Fig. 3) and are likely to be intra-priming artifacts produced by long-read protocols (Tardaguila *et al.*, 2018). In such cases, having an aligner that is not limited to aligning to only gene regions is preferred. We observed that this is resolved by using uLTRA as a wrapper around minimap2. Overall, our experiments on simulated, synthetic and biological data indicated that uLTRA_mm2 (i.e. uLTRA as a wrapper around minimap2) produced the most favorable alignments at the cost of a slightly higher runtime on the biological sequencing datasets.

We noted that the computational bottleneck of uLTRA is the MEM finding using slaMEM, particularly, it is accountable for over 60% of the total runtime when parallelizing over many cores. As this is a modular step in the algorithm, we will continue to explore faster alternatives to generate MEMs, or alternatively, to use other seeding approaches such as minimizers (Roberts *et al.*, 2004) or strobemers (Sahlin, 2021). While the chaining coverage cannot be computed exactly under those approaches, they may still be suitable as seeding approaches for our algorithm.

## 5 Conclusion

We present a new splice alignment algorithm and its implementation uLTRA. Our method models splice alignment as a two-pass collinear chaining problem with a novel exon chaining formulation. Our analysis highlights some of the challenges with splice alignment and the current state-of-the-art approaches. We show that uLTRA substantially improves splice-alignment accuracy of long RNA-seq reads using simulated, spike-in and biological datasets. On an Alzheimer Brain Isoform Sequencing dataset from PacBio, we demonstrate several examples where uLTRA aligns reads to previously annotated and novel isoform structures that the other aligners did not detect. This highlights the immediate utility that uLTRA has when profiling a new transcriptome. Furthermore, uLTRA can be used both as a stand-alone aligner and as a wrapper around minimap2 to handle reads aligning to unannotated regions.

## Supplementary Material

btab540_Supplementary_Data
