# Supplementary material for: Accurate spliced alignment of long RNA sequencing reads
Source: Bioinformatics. 2021 Jul 24;37(24):4643–51. doi: 10.1093/bioinformatics/btab540 (PMC8665758; doi:10.1093/bioinformatics/btab540)
Supplement: btab540_Supplementary_Data [file btab540_supplementary_data.pdf]

Supplementary data

Note A: Alignment parameters

uLTRA

We ran uLTRA (v 0.0.2, commit 04290f6) for all five datasets.

Indexing

We ran indexing, which consists of two steps `prep_splicing` and `prep_seqs` as follows.

uLTRA prep_splicing --disable_infer GTF_annotation outfolder

uLTRA prep_seqs ref_fasta outfolder --min_mem X

Where X=17 for DROS, SIRV, and the simulated read datasets, and X=20 for ALZ and ENS data. Also, the prep_splicing method sets the following default parameters: --flank_size 1000, --mask_threshold 200, --small_exon_threshold 200.

Alignment

Within the algorithm, uLTRA sets the --ont parameter for the DROS, SIM_ANN, and SIM_NIC datasets to encompass an error rate of 5-12%, and --isoseq parameter for the SIM_ENS and ALZ dataset for lower error rates. The --ont parameter sets minimum MEM size of 17 and MAM alignment minimum accuracy of 0.6, while --isoseq sets minimum MEM size of 20 and MAM alignment minimum accuracy of 0.8. uLTRA uses slaMEM (Fernandes and Freitas 2014) to find MEMs, edlib (Šošic and Šikic 2017) to find MAMs, and parasail (Daily 2016) to perform final alignment to exons. We use alignment with parasail in semi-global mode with alignment penalties chosen as match:2, mismatch:-2, gap open: -3, gap extend: -1.

uLTRA also sets the following parameters related to the number of alignments to report --dropoff 0.95, --max_loc 5.

Minimap2

We ran minimap2 (H. Li 2018) (v2.17-r974-dirty, GitHub commit c9874e2). We used -k 14 for PacBio Alzheimer dataset and -k 13 for the other datasets to index the reference genomes. For alignment with minimap2, we specified parameters --eqx -t 19 -ax splice -k14 -G 500k for the PacBio Alzheimer dataset, and --eqx -t 19 -ax splice -k13 -w 5 -G 500k for the rest of the datasets. The -k and -w parameters were set to 13 and 5, respectively, to improve accuracy over default parameters. We observed a 0.25%, 0.5%, and 0.72% increase in the number of correct alignments over default parameters on SIM_ENS, SIM_ANN, and SIM_NIC. We also increased the -G parameter from the default value of 200,000 to 500,000 in all datasets except for SIRV. The parameter -G controls maximum intron length. We observed that minimap2 missed valid high-quality mapped isoforms in the ALZ dataset with introns larger than 200,000 (e.g., 10 reads that uLTRA found to transcript ENST00000555571.5 with an intron of length 387,219nt).

Finally, minimap2 sets a special penalty score for non-canonical junctions. We set the non-default parameters --splice-flank=no --secondary=no -C5 on the SIRV dataset as suggested in the minimap2 documentation and discussed in issue 99 in the minimap2 repository since SIRV isoforms do not honor the canonical AG-GT junction to the same extent as biological data. We observed a drastic improvement with the setting --splice-flank=no compared to default splicing mode. With original splicing parameters, minimap2 only mapped reads as FSM to 45 unique isoforms.

deSALT

We ran deSALT and deSALT_GTF (v1.5.6, commit 733831b) on all datasets besides the ALZ dataset with deSALT_GTF for which the v1.5.6 version returned an error. On this dataset, we used an earlier version (v1.5.5). We indexed the genomes with default parameters, as suggested in (Liu et al. 2019). We ran deSALT alignment with parameters -d 10 and -s 2 for all datasets, as (Liu et al. 2019) used these settings for both ONT and PacBio Iso-Seq data. We used the parameter -l 14, which lowers the seeding kmer size from the default of 15 and is supposed to increase accuracy at the cost of runtime. We also increased the --max-intron-len parameter from the default value of 200,000 to 500,000 in all datasets except for SIRV. The parameter --max-intron-len controls maximum intron length, and we observed, similarly to minimap2, that deSALT missed valid isoforms with intron larger than the default value of 200,000.

Similar to minimap2, deSALT sets an individual penalty score for non-canonical junctions. We set the parameter --noncan to 4 instead of the default of 9 on the SIRV data, and, similarly to minimap2's results, and we observed a substantially improved accuracy over default parameters. With original splicing parameters, deSALT only mapped reads as FSM to 47 unique isoforms.

Graphmap2

We could not install Graphmap2 on one Linux system and we received segmentation faults on another system. These issues were reported (GitHub issues 17 and 18) for commit 11815ed on github.

Note B: Simulated data

Simulating from annotated transcripts

We considered using already existing read simulators (NanoSim (Yang et al. 2017), DeepSimulator (Y. Li et al. 2018), SimLord (Stöcker, Köster, and Rahmann 2016), and SNaReSim (Faucon et al.(“SNaReSim: Synthetic Nanopore Read Simulator - IEEE Conference Publication” n.d.)) before implementing a transcriptomic read simulator. However, they are genomic read simulators and cannot easily be modified to simulate full-length transcript reads.

In our simulations, we simulate transcripts from 234,207 distinct ENSEMBL cDNA sequences from the human reference. We sample reads uniformly from the transcripts. Each sampled transcript is then subject to sequencing errors. At each position in the transcript, we enter an error state with a probability of 0.05. If the simulator enters an error state, it chooses a deletion state with a probability of 0.5, substitution with a probability of 0.3, and insertion with a probability 0.2. In the deletion and insertion states, the length of the insertion and deletion is simulated from a geometric distribution with a parameter of 0.5. This model gives an average error rate of 8.6%.

Simulating novel transcripts

We used the GTF annotations for this simulation. We select all genes with four or more exons that have non-overlapping genomic coordinates. For each transcript we simulate from such a gene, we include the first and last exon with probability 1, but any internal exon with a probability of 0.5. This probabilistic inclusion of exons gives isoforms. We check if the transcript matches an already annotated isoform. If not, we keep it in the simulation. To keep the number of transcripts equal to the annotated number of transcripts for each gene, we simulate as many novel transcripts as the gene had annotated transcripts (if the number of possible combinations permits).

Evaluation of simulated data

For the simulated data, we have the true genome annotations of each exon in the transcript. A read is therefore classified as *correct* if the read is aligned to all the correct exons, and all of the exon alignments have an offset of fewer than 15 nucleotides to true annotation coordinates. An alignment is *inexact* if the read is aligned to the correct exons, but at least one junction offset is more than 15 nucleotides. An alignment is classified as having an *exon difference* if the read alignment is missing one or more exons, or a segment of at least 15 nucleotides is aligned to a genomic location not included in the set of true exons (i.e., appearing as a false exon). An alignment is classified as an *incorrect location* if it aligns to a genomic location not overlapping with the correct annotation. In our classifications above, we chose 15bp as the threshold because we did not observe deletions or insertions longer than 15 nucleotides in the simulated data. Therefore, an offset larger than 15 nucleotides indicates a misalignment rather than an actual deletion or insertion of this size in the read, causing a junction offset.

As a 15 nucleotide threshold may be excessive when identifying novel splice sites, we also compared alignment results in a more stringent setting. Here, we do not allow any offset in alignment to the true splice site for an alignment to be classified as correct.

Note C: SIRV analysis

Data

The SIRV dataset was downloaded from ENA under project accession number PRJEB34849. This dataset was processed with pychopper (https://github.com/nanoporetech/pychopper, commit 6dca13d) which detects reads covering the entire transcript (full-length reads) by identifying barcodes present in the reads. Pychopper was run with default parameters.

The SIRV dataset consists of 68 synthetic transcripts from 7 different loci sequenced with ONT R9 technology (see (Sahlin and Medvedev 2021) for details). The transcripts from each locus differ in their splicing pattern. Eight out of the 68 transcripts contain only one exon, and therefore do not have a splice site. Furthermore, two isoforms SIRV701 and SIRV705 have identical splice sites and differ only in a 2nt offset in both the transcription start and stop site. Therefore we used 59 isoforms with distinct splice sites to investigate alignment performance around splice sites. The ONT SIRV dataset was constructed so that the isoforms should have roughly the same abundance in the sample (the SIRV E0 mix). While sequencing depth bias can cause significant differences in read depth due to the isoforms having different lengths, with the given depth of 1.4 million reads, we expect all the isoforms to occur in the sample.

Analysis

In this dataset, as the sequenced isoforms are known, we have a complete isoform annotation. We computed all alignments that had perfect matching splice sites to the annotations and denoted these reads as Full Splice Matches (FSM) following the notation in (Tardaguila et al. 2018). With the SIRV dataset, we have the properties of real ONT sequencing errors and genes, each expressing several known isoforms. The downside with SIRV data is that it does not represent the sequence complexity of a genome. For minimap2 and deSALT, we used non-default alignment parameters not to penalize non-canonical splice sites as much as in biological data. After this modification, we observed substantially improved alignment performance over default parameters (for details, see Suppl. Note A).

While sequencing bias may distort the read coverage per isoform and produce a dataset with different coverage distribution to what is present in the sample, the E0 mix contains transcripts at roughly equal abundances. In large, we observe similar distribution in the number of FSM alignments per isoform (Fig. S3) for all the aligners, but minimap2, minimap2_GTF, and deSALT produce fewer FSM alignments compared to deSALT_GTF, uLTRA, and uLTRA_mm2 across most isoforms. A notable difference is that minimap2 only aligns FSM reads to 54 unique isoforms, even after employing specific alignment parameters for SIRV data (Suppl. Note A). In comparison, deSALT and uLTRA in both settings were able to align FSM reads to all 59 unique isoforms.

There are some exceptions that we will now discuss. Firstly, both deSALT and minimap2 in annotation-free and annotation-provided settings align substantially fewer reads to SIRV503 than uLTRA and uLTRA_mm2. SIRV503 contains an 8nt long exon that deSALT and minimap2 do not align to in the large majority of reads containing the exon. Secondly, minimap_GTF does not align any reads to SIRV511, SIRV708, SIRV304, SIRV403, and SIRV408, while the annotation-free version of minimap2 does. Instead, we can see that minimap_GTF aligns a substantially larger fraction of reads to, e.g., SIRV506. Without setting specific alignment parameters for this dataset (Suppl. Note A), we observed that deSALT_GTF did not align any reads to SIRV511 and SIRV708. Such cases show that using specific alignment parameters for non-canonical splice sites may introduce alignment bias from overfitting to specific isoforms. Thirdly, comparing the two reference-free alignment methods minimap2 and deSALT, we observe that deSALT produces substantially fewer FSM alignments to minimap2 across most isoforms.

Overall, we observed more evenly distributed FSM alignment across the SIRV isoforms with both uLTRA and uLTRA_mm2. While there is no ground truth for this dataset, an equal abundance of isoforms is expected in this dataset from the design of the SIRV E0 mix. Furthermore, the number of FSM isoforms between uLTRA and uLTRA_mm2 alignments stays consistent.

Note D: Biological datasets processing

The ALZ dataset was downloaded from https://downloads.pacbcloud.com/public/dataset/Alzheimer2019_IsoSeq/. This dataset has been processed with the standard Iso-Seq bioinformatics pipeline (SMRTlink 8.0 "IsoSeq" protocol) to contain only full-length reads. The DROS dataset was downloaded from ENA under project accession number PRJEB34849. This dataset was processed with pychopper (https://github.com/nanoporetech/pychopper, commit 6dca13d) using default parameters to identify full-length reads.

Note E: Alignment concordance

We first looked at a relaxed measure of alignment concordance. We define a read to have globally concordant alignments between two methods if the two alignments have a non-zero overlap on the genome (based on the start and stop coordinates). Note that this definition only captures discordance if the read aligns to different genes, not smaller differences around exons. A caveat with this definition occurs when measuring alignment concordance between more than two aligners. An alignment spanning positions A to C in one alignment may overlap with two disjoint alignments A to B and B+1 to C. In this case, we treat all the alignments as discordant. Finally, there are genes with multiple identical copies on the genome. In these cases, the alignment methods may choose different alignment locations simply by randomly picking a location.

With these limitations in mind, we observed 90.3% and 98.6% of all aligned reads had concordant genomic positions in DROS and ALZ, respectively (Fig. S4). This indicates that the mapping region is largely consistent between aligners and that most of the variability occurs in alignments around exons. The lower alignment concordance for the DROS dataset may result from a higher median error rate combined with a shorter average read length. In the DROS dataset, the second-largest category was the alignment concordance between uLTRA_mm2 and deSALT_GTF (5.7%; Fig. S4A).

We also report alignment concordance broken down individually within the classes FSM, ISM, NIC, NNC, and NO_SPLICE. For the FSM, ISM, NIC classes where splice sites are known, we classify an alignment as concordant between aligners if all the splice sites are identical. For NNC and NO_SPLICE, we use genomic overlap as described above. We observed a large concordance of alignments in the categories FSM, ISM, and NO_SPLICE and a slightly lower concordance for the categories NIC and NNC in both DROS (Fig. S5) and ALZ (Fig. S6) datasets. However, the NIC and NNC categories contain fewer reads (Fig. 3).

Note F: Isoforms uniquely detected by uLTRA

FSM isoforms uniquely found by uLTRA

On the DROS dataset, uLTRA_mm2 aligned 338 FSM reads to 104 distinct isoforms (0.8% of total distinct isoforms) that minimap2 and deSALT_GTF did not align to. Of these isoforms, 7 had more than ten reads aligned, while most other isoforms had a coverage of 1-10 reads (Fig. S8A). For the ALZ dataset, uLTRA aligned a total of 9,130 FSM reads to 571 distinct isoforms (1.6% of total distinct isoforms) that minimap2 and deSALT_GTF did not align to. A total of 109 of these isoforms had more than 10 reads aligned, while most other isoforms had a coverage of 1-10 reads (Fig. S8).

We manually inspected a subset of the more abundant uniquely predicted isoforms by uLTRA_mm2 for the ALZ dataset using IGV (Robinson et al. 2011). We observed that some of these isoforms contained small exons (<10nt) that after manual inspection appeared correctly aligned to (Fig. S9). However, deSALT_GTF and minimap2 agreed on a different splicing structure, with the small exons put as an insertion or substitutions in the 5’ or 3’ ends of upstream or downstream exons. These alignments would show up as concordant between deSALT_GTF and minimap2_GTF in our previous analysis, although they are unlikely to be correct. The isoforms in Figure S9 come from the genes AP2, APBB, HNRNPM, and DCTN2, which come from gene families that have appeared in studies related to Alzheimer’s disease (Tian et al. 2013) (Tanahashi and Tabira 1999) (Geuens, Bouhy, and Timmerman 2016) or other neurodegenerative disorders (Boland et al. 2018). All of these genes are supported by more than 100 reads and have perfect alignment across the junctions in uLTRA_mm2’s alignments.

In addition, we highlight another case of a potential subtle misalignment (Fig. S10) that makes the best fit FSM isoform go undetected. This potential misalignment is caused by using GT-AG specific alignment penalties and causes 500 reads to support a GT-AG splice junction in deSALT_GTF and minimap2. In this example, uLTRA_mm2's alignments support a GC-AG junction. While we have no ground truth, and an insertion of one nucleotide near the splice site is plausible, uLTRA_mm2's alignments best fit the data (omitting prior belief of GT-AG junction) and also support a previously annotated isoform. The PRNP gene has also been studied in Alzheimer's disease (Bagyinszky et al. 2019).

Finally, we illustrate an example (Fig. S11) of an instance of 161 reads where all three aligners have discordant alignments, caused by a segment of 9nt from a transcript from the SPOCK

gene, which has also appeared in studies on neurodegenerative disorders (Charbonnier et al. 1997). Here, uLTRA_mm2 and deSALT_GTF align the 9nt portion of the read corresponding to two different exons while minimap2 does not align this region. Both uLTRA_mm2 and deSALT_GTF alignments are FSM but to different isoforms, and both upstream and downstream junctions are GT-AG. With this information, it is ambiguous as to which alignment is the correct one.

NIC isoforms uniquely found by uLTRA

We also observed that uLTRA_mm2 aligns more reads as NIC alignments compared to deSALT_GTF and minimap2 (Fig. 3, Fig. S5C, and S6C). Many of these NIC reads could be spurious due to inaccuracies in uLTRA_mm2 alignments when both upstream and downstream flanks of the junction contain the same nucleotide. We looked at the most abundant NIC uniquely aligned to by uLTRA_mm2, a transcript from the MBP gene (Fig. S12; predicted by 943 reads). uLTRA aligned reads to this NIC because of the homopolymer length difference of C's in the reads, together with that both the upstream and downstream junction contained C's. However, deSALT_GTF and minimap2 always aligned to the CT-AC junction by creating insertions of C at downstream junctions if needed (matching an FSM), while uLTRA_mm2 chooses a CT-TA junction for the reads where the homopolymer length was four cytosine nucleotides (creating a NIC). It is ambiguous as to what is the correct isoform in this example.

As for the uniquely predicted FSMs, the NICs also contained predictions with small exons (in total 50 unique NIC isoforms have exons smaller than 20nt). We manually inspected some of the more abundant predictions of which, similarly to the FSMs with small exons, the data supports their correctness (Fig. S13 A-C). The three isoforms presented in Fig. S13 are highly supported isoforms from the MICU1, SEPTIN7, and APBB1 genes and are, furthermore, a novel with respect to the Gencode v34 annotation and have appeared in studies on Alzheimer’s disease (Wang et al. 2018) (Calvo-Rodriguez et al. 2020) (Tanahashi and Tabira 1999).

Note G: Runtime and memory usage

We used a 128Gb memory node with 20 cores. We tested the tools using both 4 and 19 cores (leaving one core for the main process) to study parallelization performance. We measured user time (total time from start to finish) and peak memory usage (highest memory usage across the program lifetime).

Using 4 cores, we observe that deSALT is the fastest tool except on the largest dataset (ALZ) where minimap2 is the fastest (Table 2). The relative runtime difference between uLTRA and

deSALT decreases with the organism's size and the number of reads on our datasets. For example, on the two smaller datasets SIRV and DROS, deSALT is about 6 times and 4 times faster, respectively. While on the largest datasets, SIM_NIC and ALZ, deSALT is about 2 times and 1.6 times faster, respectively (Table 2). uLTRA has a similar or faster runtime than minimap2 on ENS, SIM_ANN, and SIM_NIC, respectively, but is slower on the other datasets. Using uLTRA as a wrapper around minimap2 increases the runtime slightly, but it is substantially less costly than running both aligners separately. For the ALZ dataset, running uLTRA_mm2 is only about 8% slower than only running uLTRA. Overall, while uLTRA and uLTRA_mm2 have a slightly larger runtime than minimap2 and deSALT, the practical difference, particularly for the larger datasets, is not major. When we supply 19 cores for alignment, we see similar trends as to using 4 cores. The alignment time difference between uLTRA and the other aligners is the largest for smaller datasets such as SIRV and DROS, and evens out for larger datasets SIM_NIC and ALZ (Table S1). For example, uLTRA is about 80% slower on the ALZ dataset than both minimap2 and deSALT.

A computational bottleneck is the MEM finding using slaMEM. slaMEM trades speed for memory footprint. On the DROS and ALZ datasets, the MEM finding step is accountable for 35% and 22% of the total uLTRA runtime when using 4 cores, and for over 60% of the runtime when using 19 cores.

As for memory usage during alignment using 4 cores, minimap2 and uLTRA have similar memory footprints across the three simulated datasets, but uLTRA uses about 1.7-2.6 times more memory on the biological datasets (Table S2). deSALT uses the most memory on the simulated datasets and has a memory footprint similar to uLTRA on the ALZ dataset but uses lower memory on the SIRV and DROS. When parallelizing over 19 cores, the relative memory usage between minimap2 and uLTRA stays largely the same (Table S3). However, deSALT slightly decreases its relative memory footprint compared to minimap2 and uLTRA.

For indexing, uLTRA and minimap2 are relatively fast, while deSALT is slower (Table S4). uLTRA used the smallest amount of memory (Table S5), which is not surprising as it is processing a smaller region of the genome.

Supplementary Figures

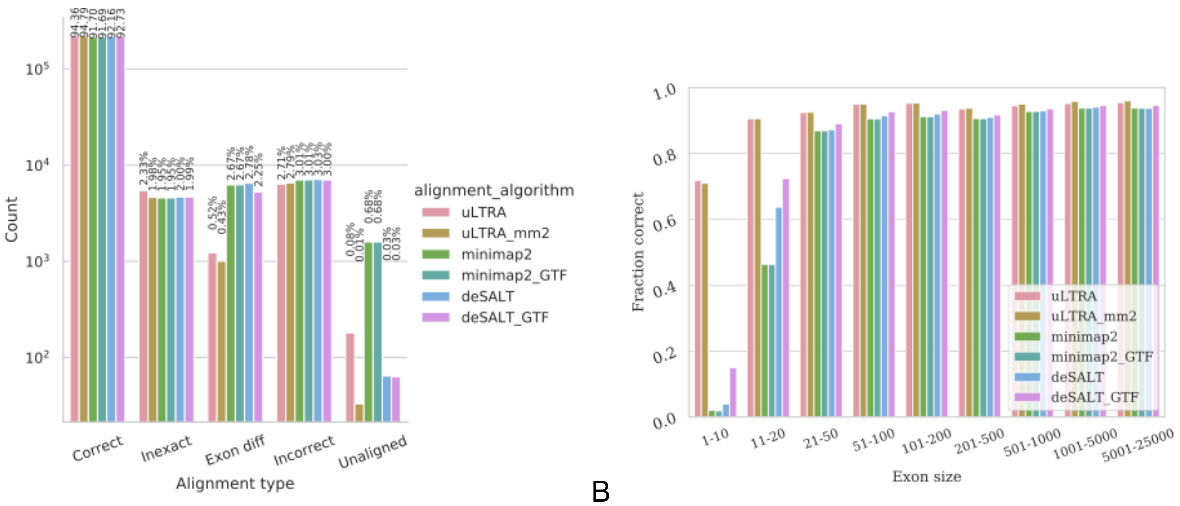

A B

Figure S1. Alignment results on simulated data for the ENS dataset. (**A**) Percentage of reads in each respective category. (**B**) The fraction of correctly aligned exons (y-axis) as a function of exon size (x-axis).

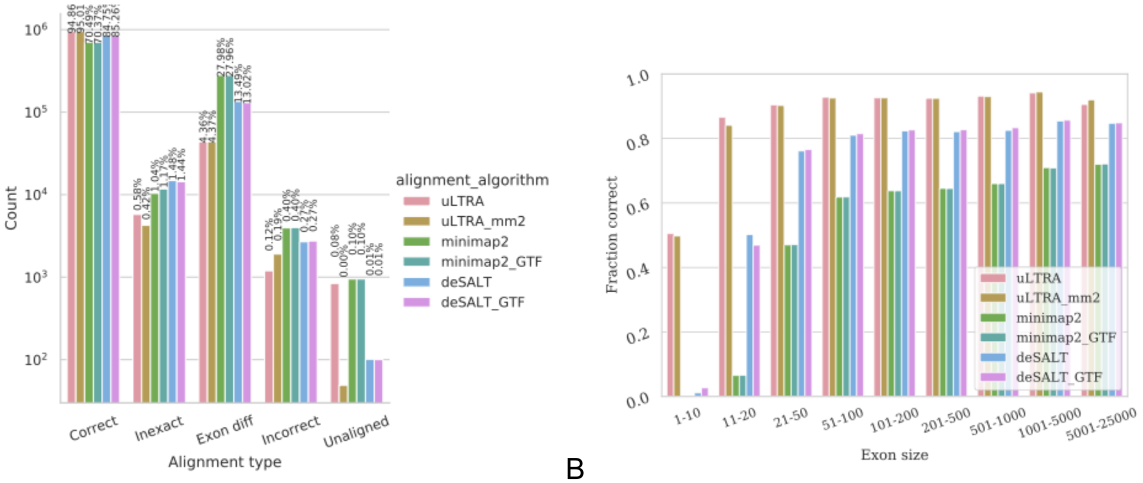

A B

Figure S2. Alignment results on simulated data for the SIM_NIC dataset. (**A**) Percentage of reads in each respective category. (**B**) The fraction of correctly aligned exons (y-axis) as a function of exon size (x-axis).

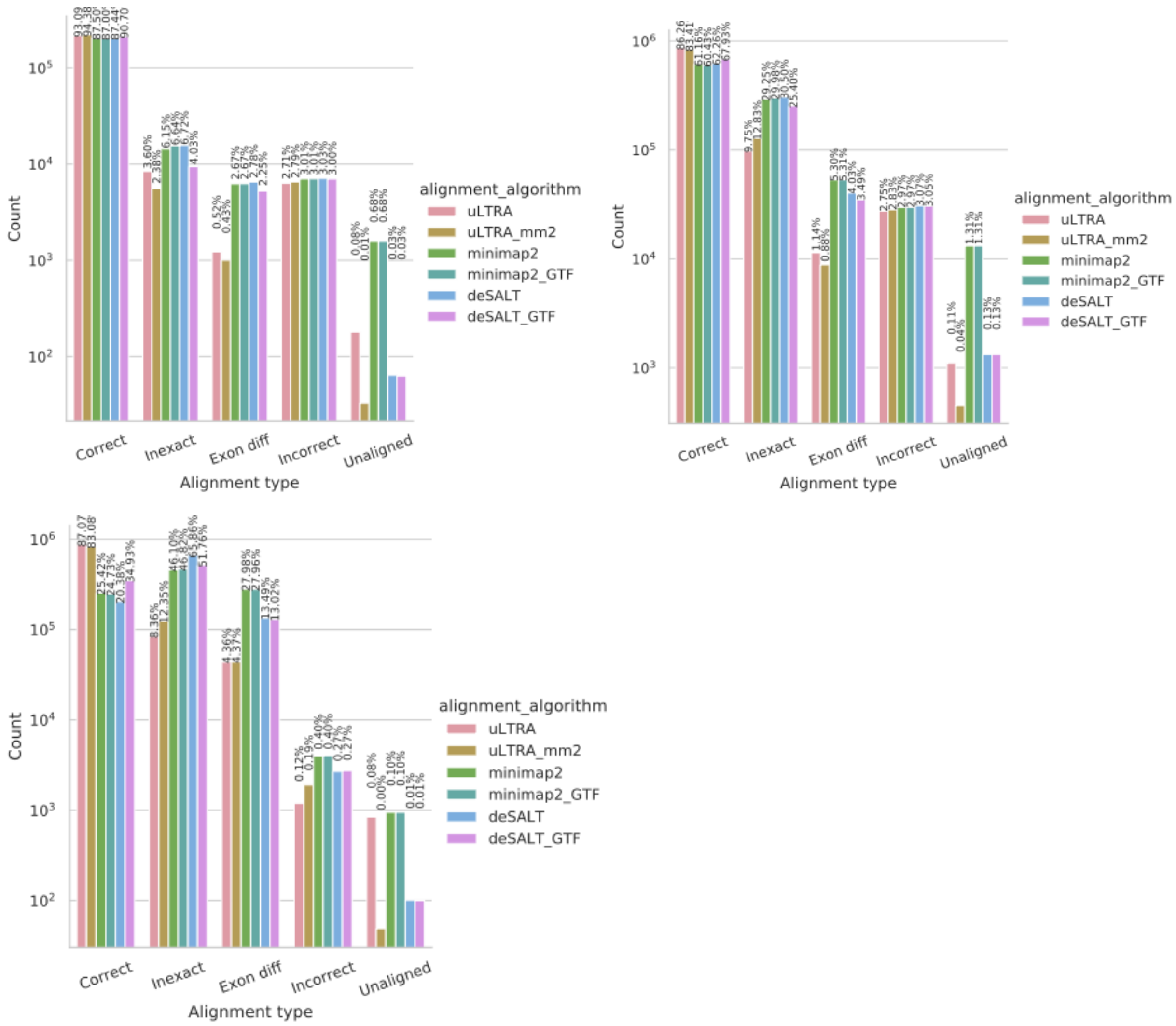

Figure S3. Alignment results on simulated data for the ENS (**A**), SIM_ANN (**B**), and SIM_NIC (**C**) dataset when applying a stringent 0 nucleotides offset threshold for an alignment to be classified as correct.

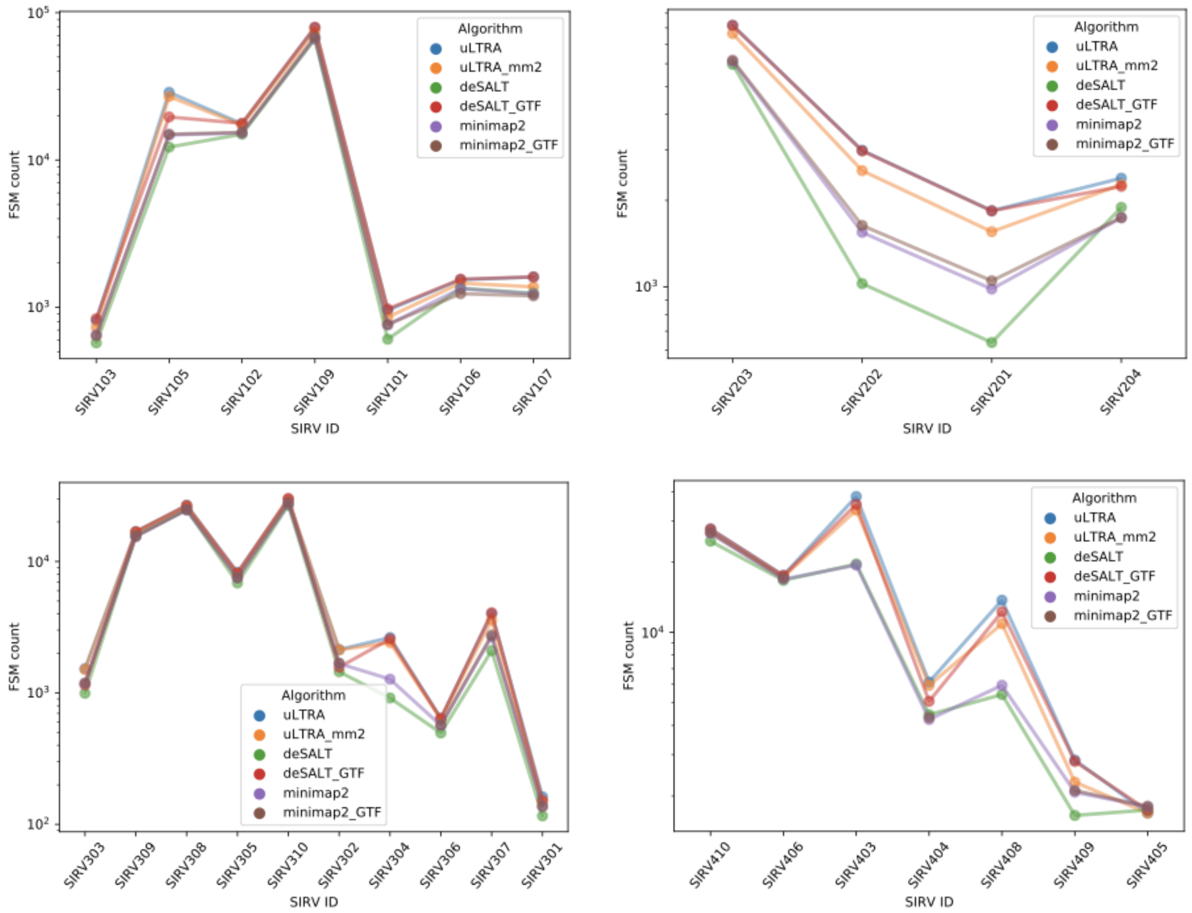
Algorithm
uLTRA
uLTRA_mm2
deSALT
deSALT_GTF
minimap2
minimap2_GTF
FSM count
SIRV ID
SIRV103
SIRV105
SIRV102
SIRV109
SIRV101
SIRV106
SIRV107
SIRV203
SIRV202
SIRV201
SIRV204
SIRV303
SIRV309
SIRV308
SIRV305
SIRV310
SIRV302
SIRV304
SIRV306
SIRV307
SIRV301
SIRV410
SIRV406
SIRV403
SIRV404
SIRV408
SIRV409
SIRV405

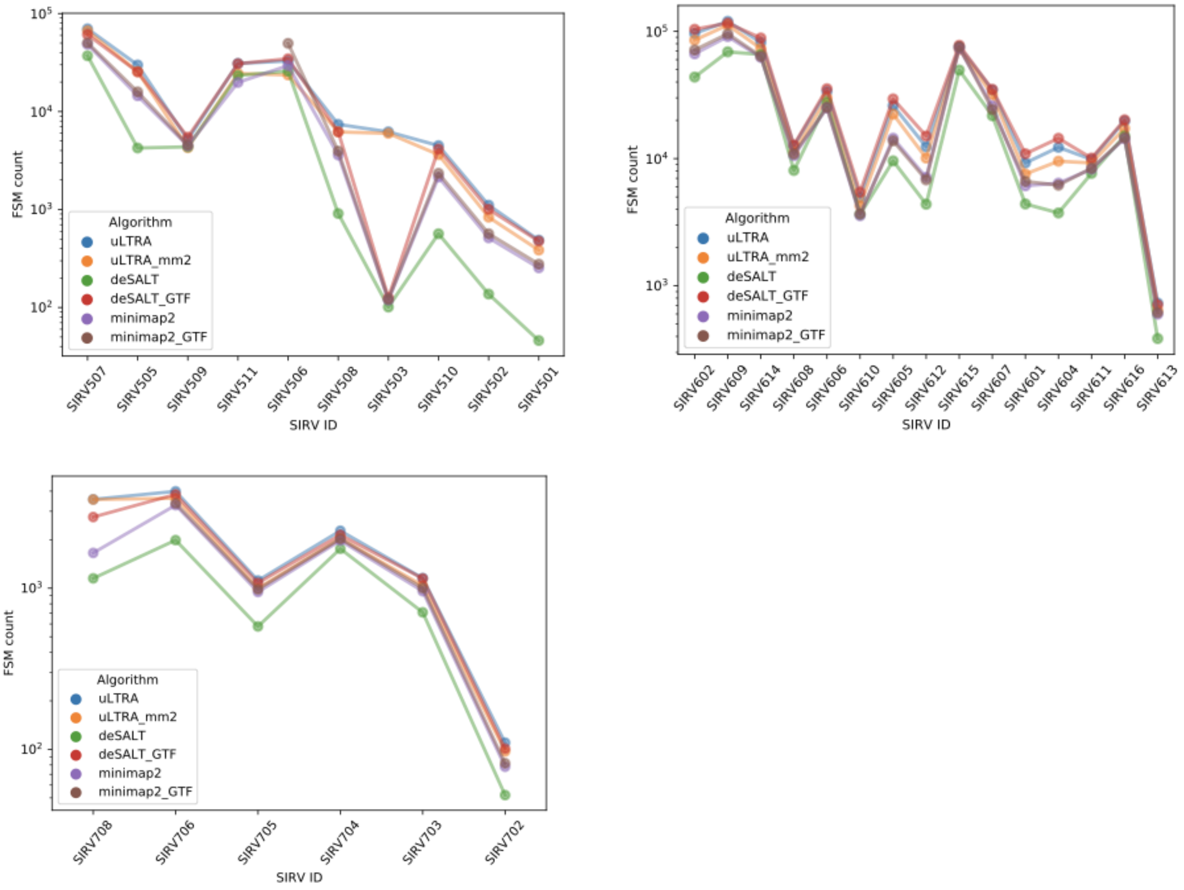

Figure S4. The number of reads annotated as FSM to each of the 59 SIRV isoforms with at least one splice site (y-axis log scale). One panel per gene loci.

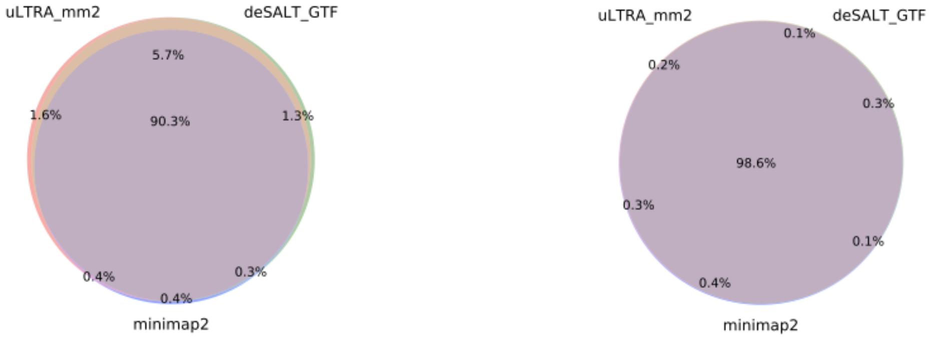

Figure S5. Alignment overlap concordance between uLTRA_mm2, deSALT_GTF, and minimap2 for DROS (A) and ALZ (B).

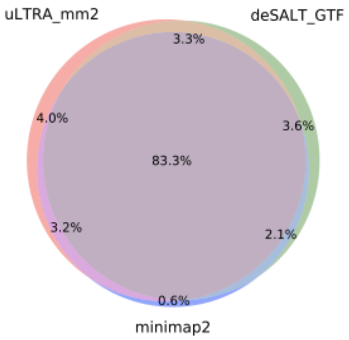

A

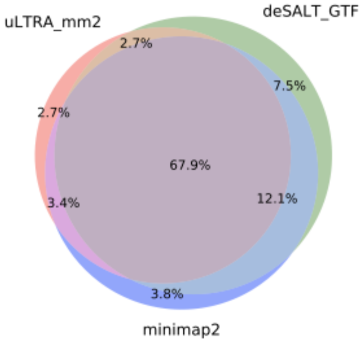

B

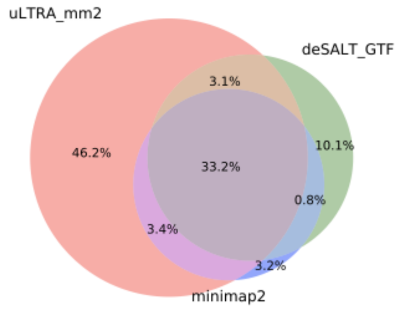

C

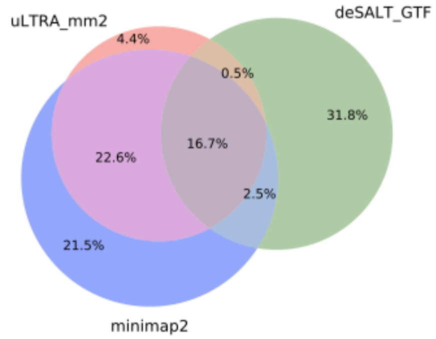

D

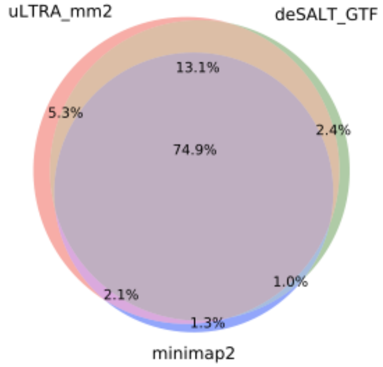

E

Figure S6. Alignment concordance between uLTRA_mm2, deSALT_GTF, and minimap2 for the different categories FSM (A), ISM (B), NIC (C), NNC (D) and NO_SPLICE (E) in DROS.

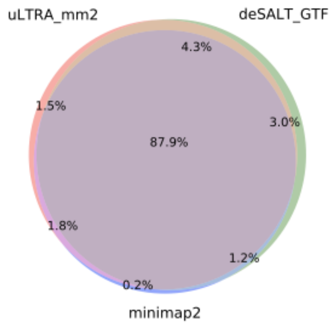

A

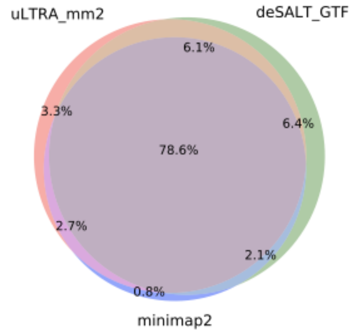

B

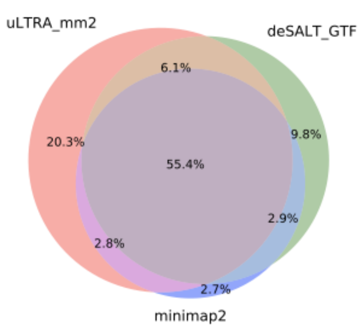

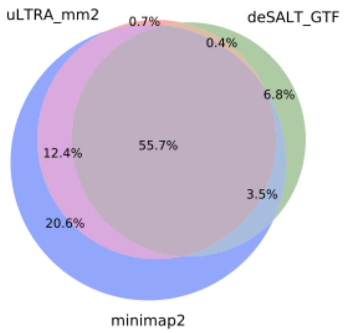

C

D

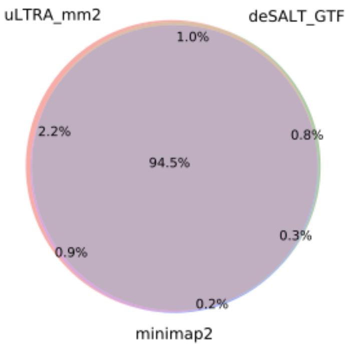

E

Figure S7. Alignment concordance between uLTRA_mm2, deSALT_GTF, and minimap2 for the different categories FSM (A), ISM (B), NIC (C), NNC (D) and NO_SPLICE (E) in ALZ.

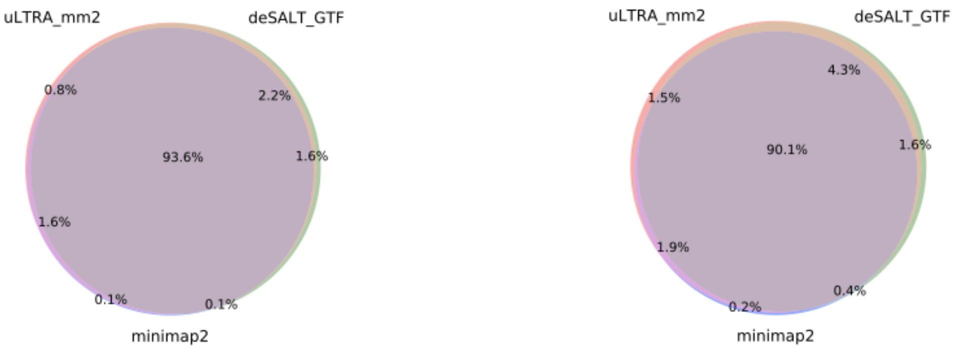

Figure S8. Concordance of unique isoforms that had FSM aligned reads for DROS (A) and ALZ (B).

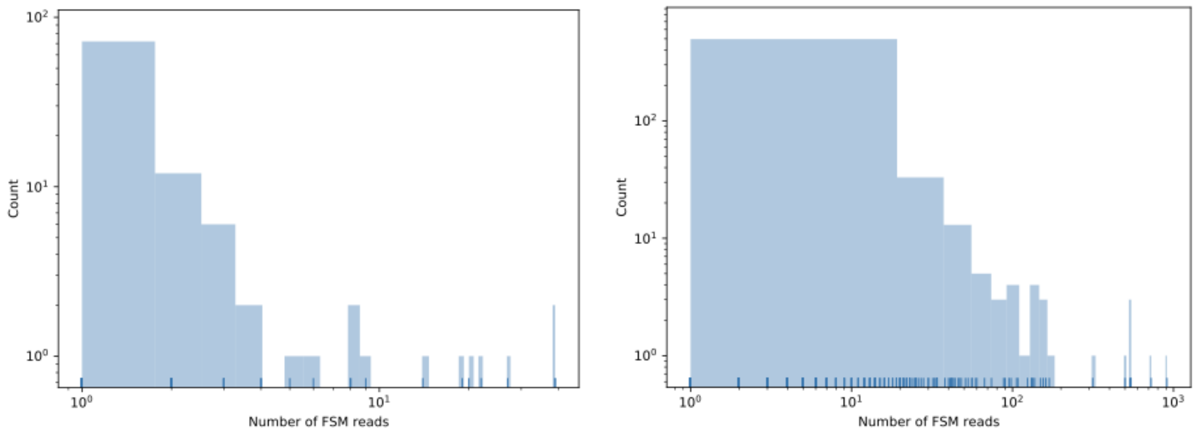

Figure S9. Histogram of the number of distinct isoform structures (y-axis) for which uLTRA_mm2 aligned FSM reads to, but deSALT_GTF and minimap2 did not. The x-axis shows the FSM read support for these isoforms. Panel (A) shows the DROS dataset and (B) the ALZ dataset.

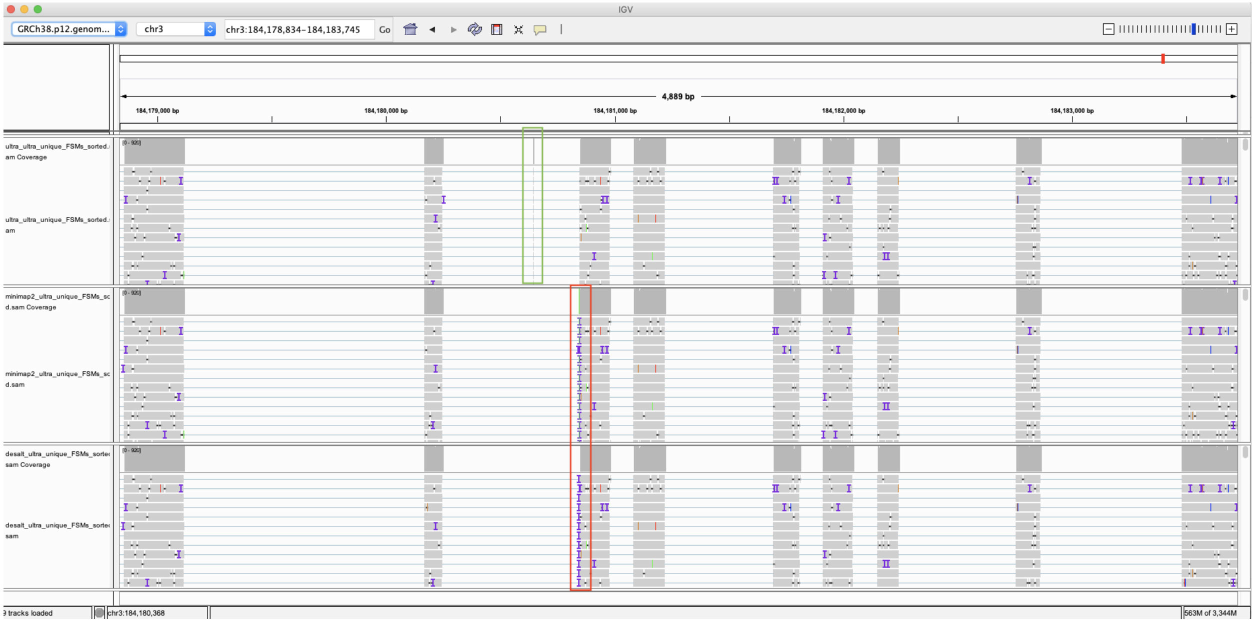

A

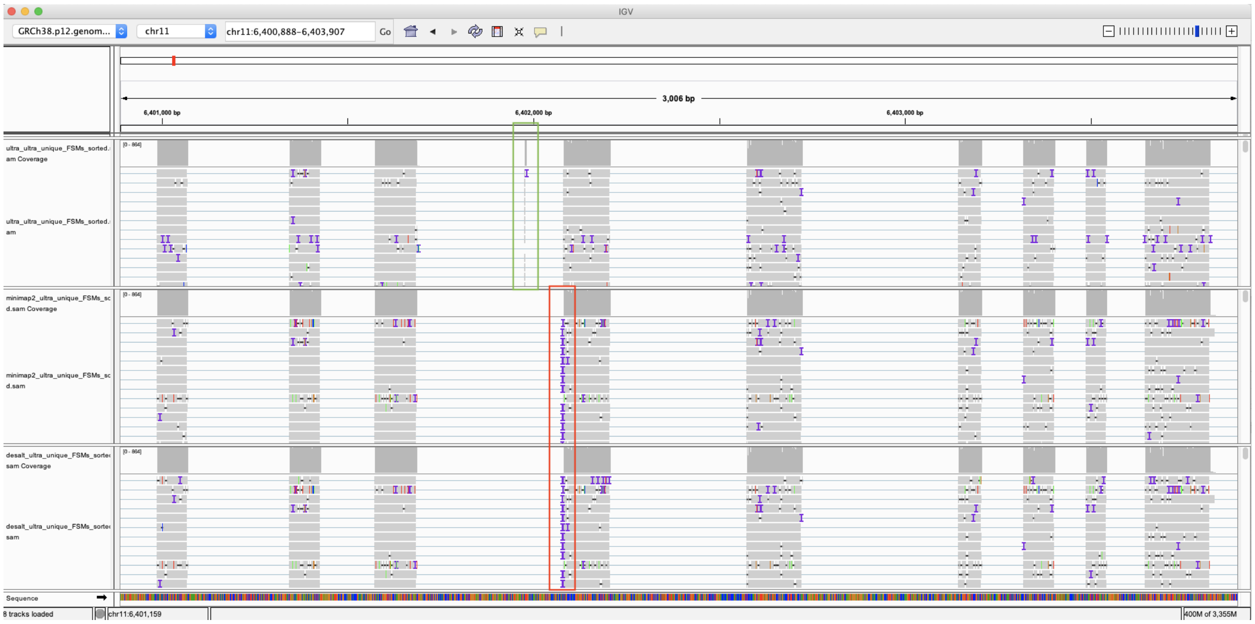

B

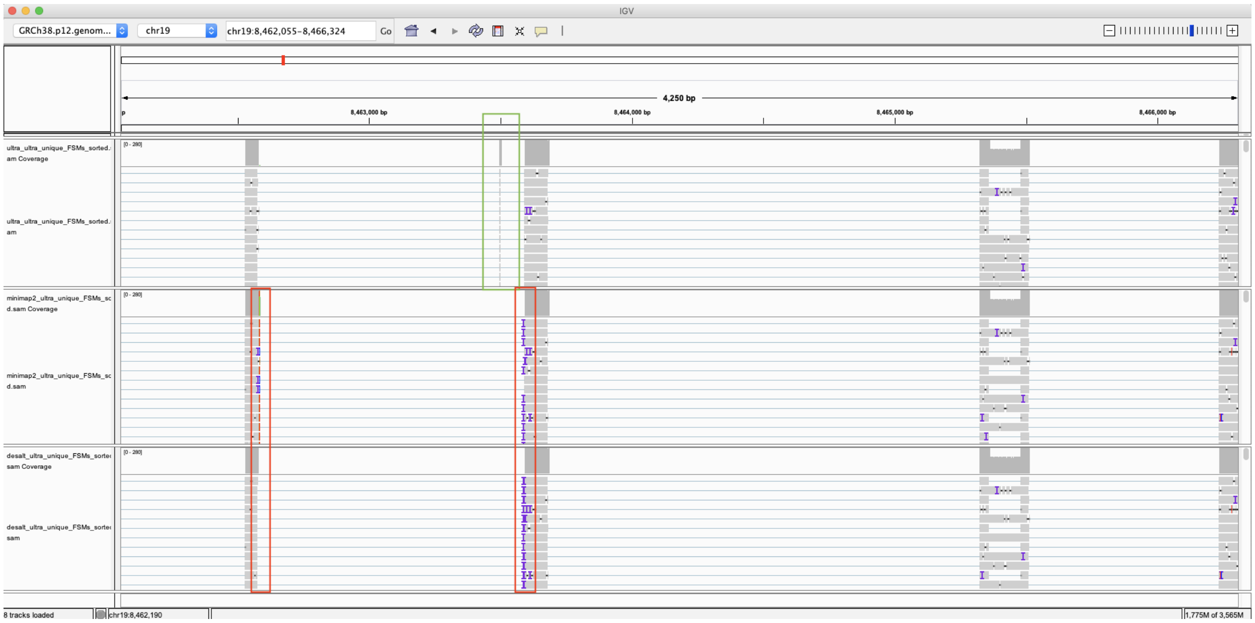

C

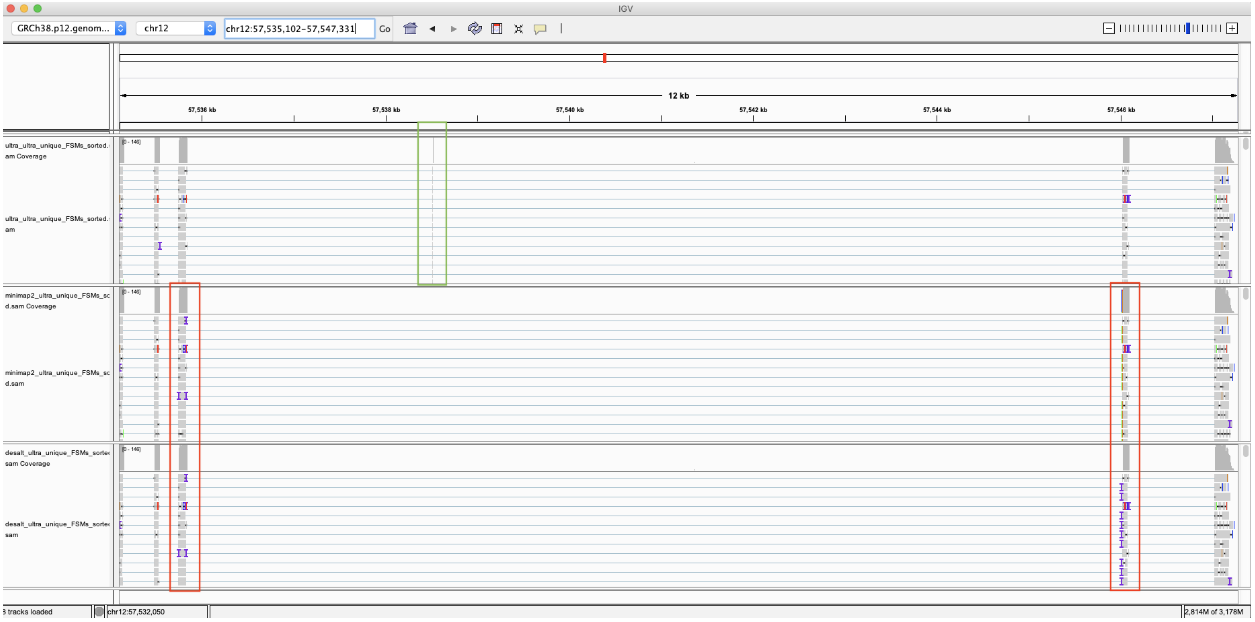

D

Figure S10. IGV tracks of FSM splice read alignments to unique FSM isoforms found by uLTRA_mm2 (first track) by aligning to small exons (6-8nt), with corresponding aligned reads of minimap2 (second track) and deSALT_GTF (third track). The green box highlights the best fit exon alignment. The red box highlights misalignments around the junctions caused by the unaligned small exons. (A) 910 reads mapping to transcript ENST00000292807.9 (AP2 gene). (B) 726 reads mapping to transcript ENST00000609360.6 (APBB gene). (C) 155 reads mapping to ENST00000325495.9 (HNRNPM gene). (D) 134 reads mapping to ENST00000543672.5 (DCTN gene).

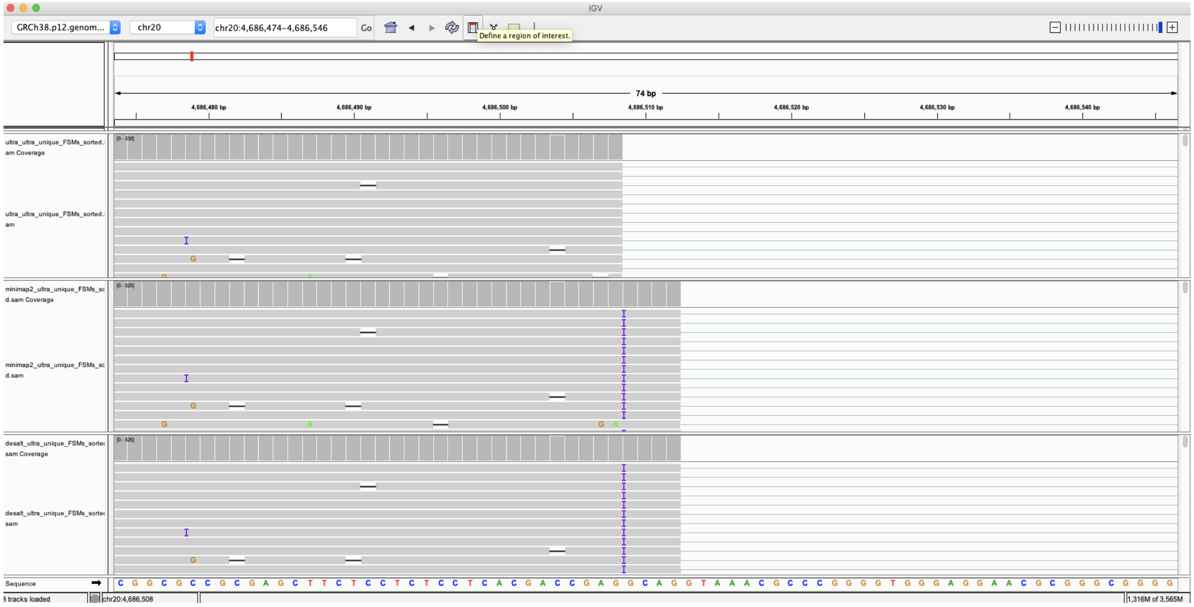

A

B
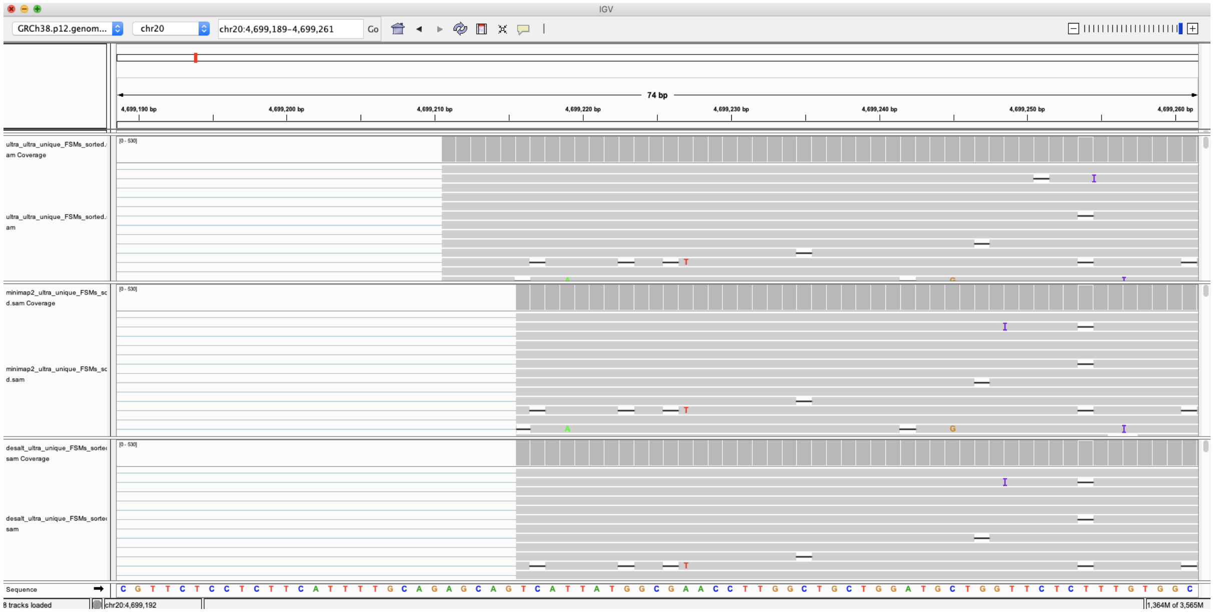

Figure S11. Potential subtle misalignment of 500 reads around a junction for minimap2 (second track) and deSALT_GTF (third track) cause structural change in splice junction. Both minimap2 and deSALT_GTF place a 5nt segment in the upstream junction (with a 1nt insertion) instead of placing the 5nt segment (full match) in the downstream junction (B). This is caused by overfitting the alignment to match a GT-AG junction with junction-specific penalties. In this gene, it appears as a small variant between the sample and the reference. Note that since the correct annotation is unknown for biological data, hence this may be a correct variation. However, uLTRA_mm2 achieves a higher identity in its alignment over the junction.

A
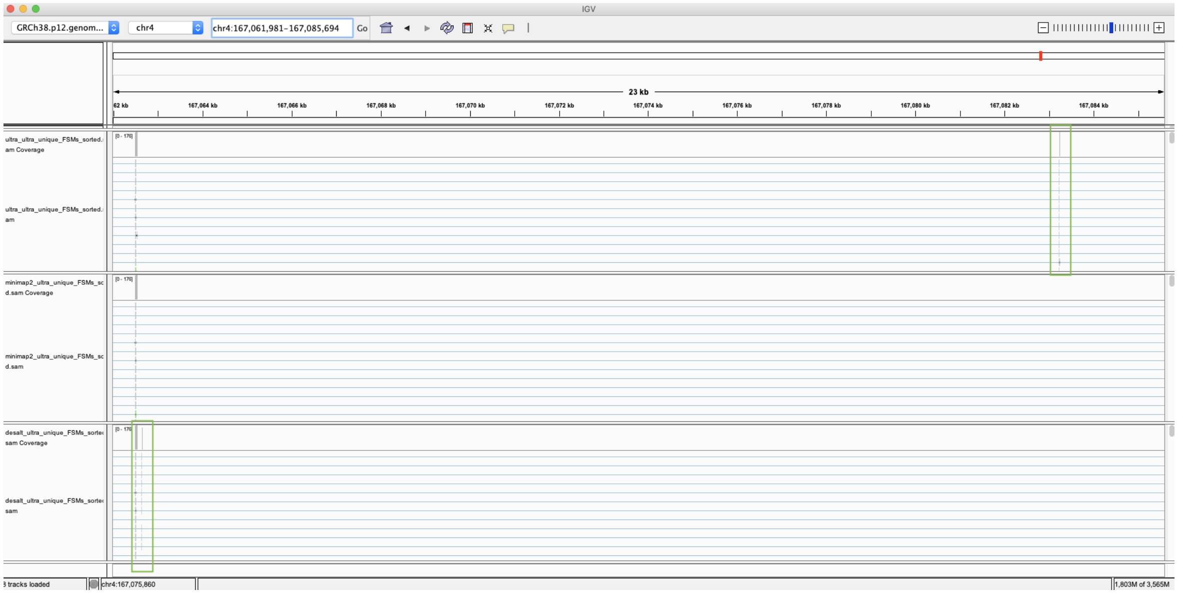

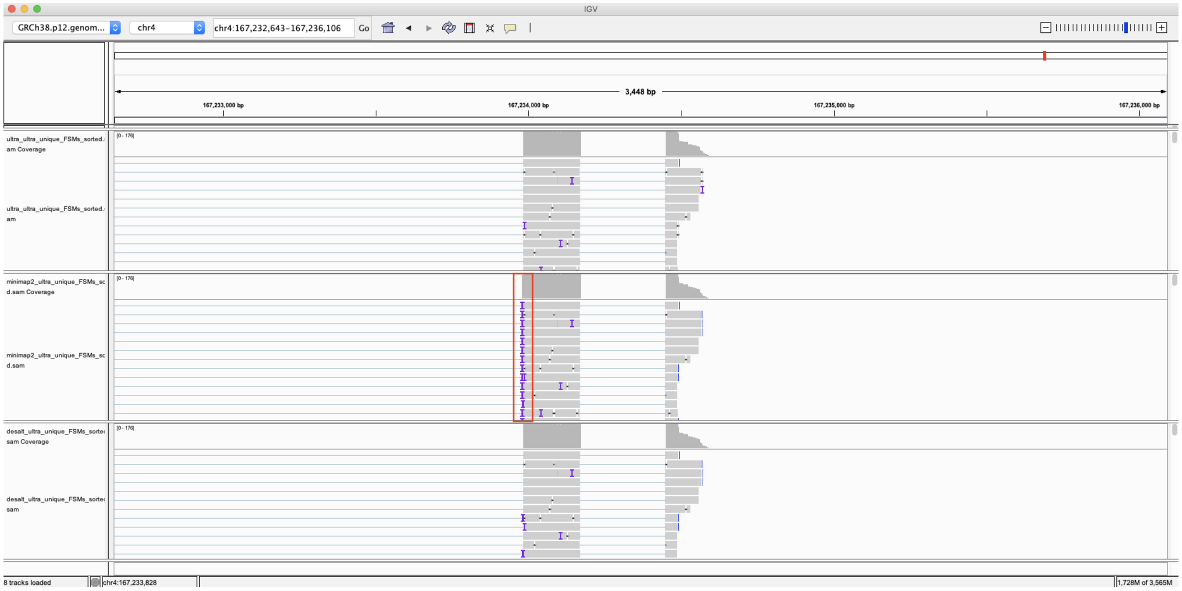

B

Figure S12. Discordant alignments of 161 reads between uLTRA (top track), minimap2 (middle track) and deSALT_GTF (bottom track) to the SPOCK gene. For these reads, uLTRA_mm2 and deSALT_GTF align a 9nt portion of the reads to two different exons (A) while minimap2 does not align this region and is instead present as an insertion in the downstream exon (B). uLTRA_mm2 chooses the upstream exon because of the deterministic implementation of taking the closest segment to the downstream hit in the traceback vector of the collinear MAM-chaining solution.

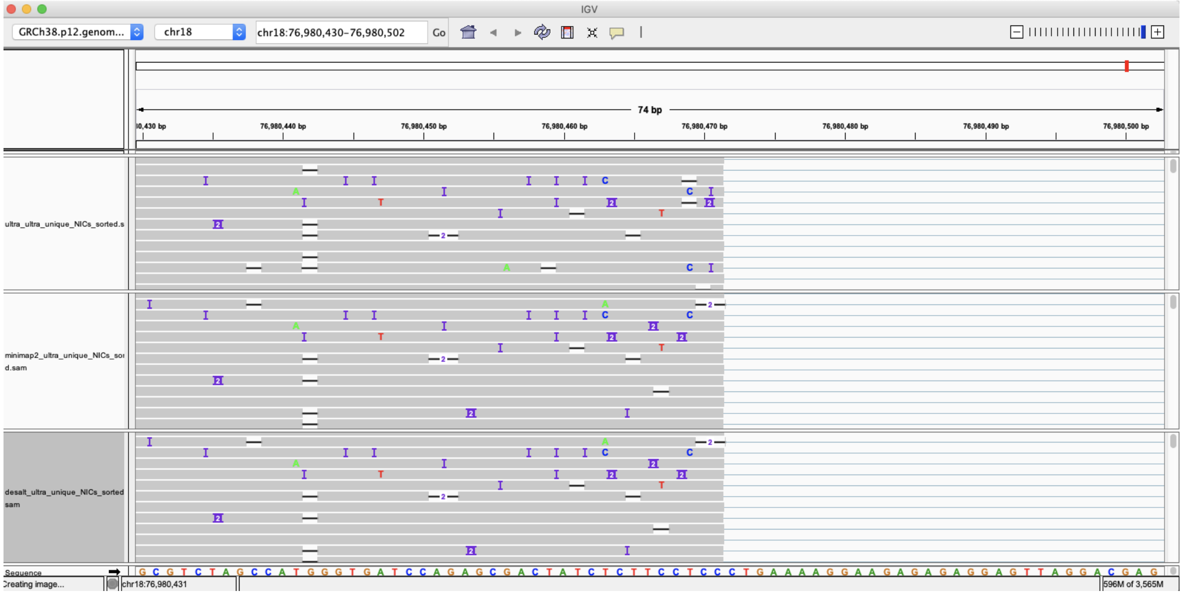

A

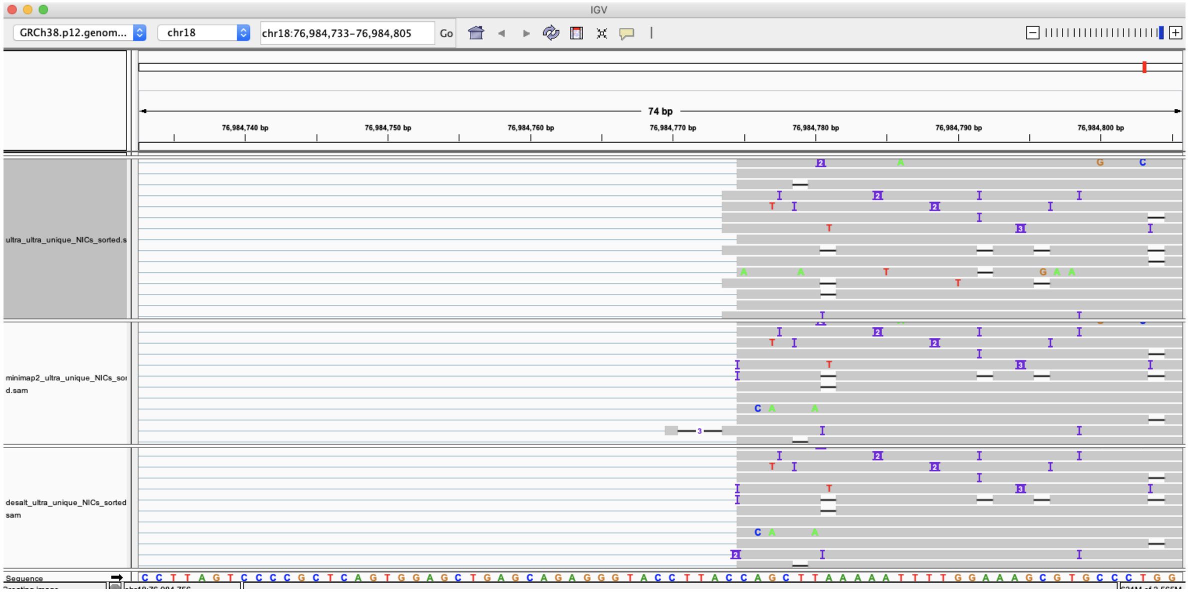

B

Figure S13. IGV tracks of splice read alignments to a junction of the MBP gene. Homopolymer stretches of C both upstream (A) and downstream (B) of the junction creates ambiguity in alignment. deSALT_GTF and minimap2 always align to the CT-AC junction by creating insertions of C at downstream junctions if needed, while uLTRA chooses a CT-TA junction for the reads with a homopolymer stretch of four Cs. The CT-AC matches an FSM transcript while the CT-TA junction creates a NIC transcript (943 reads).

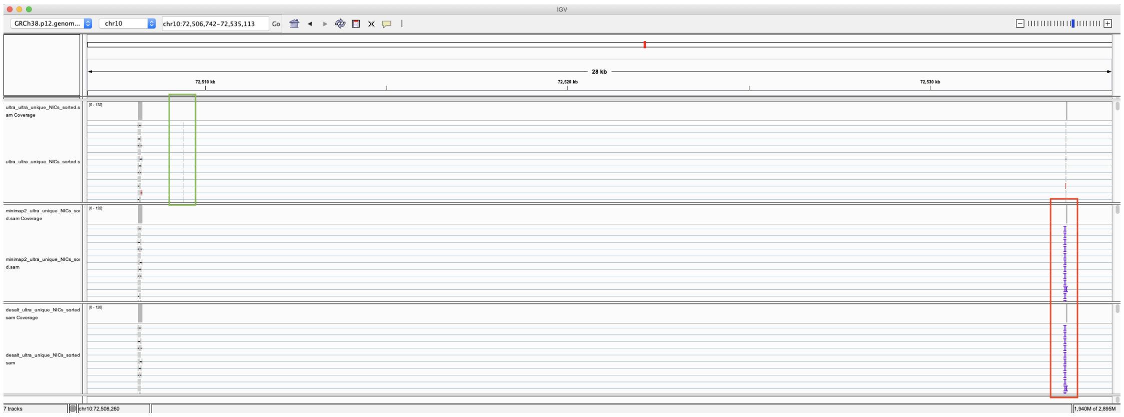

A

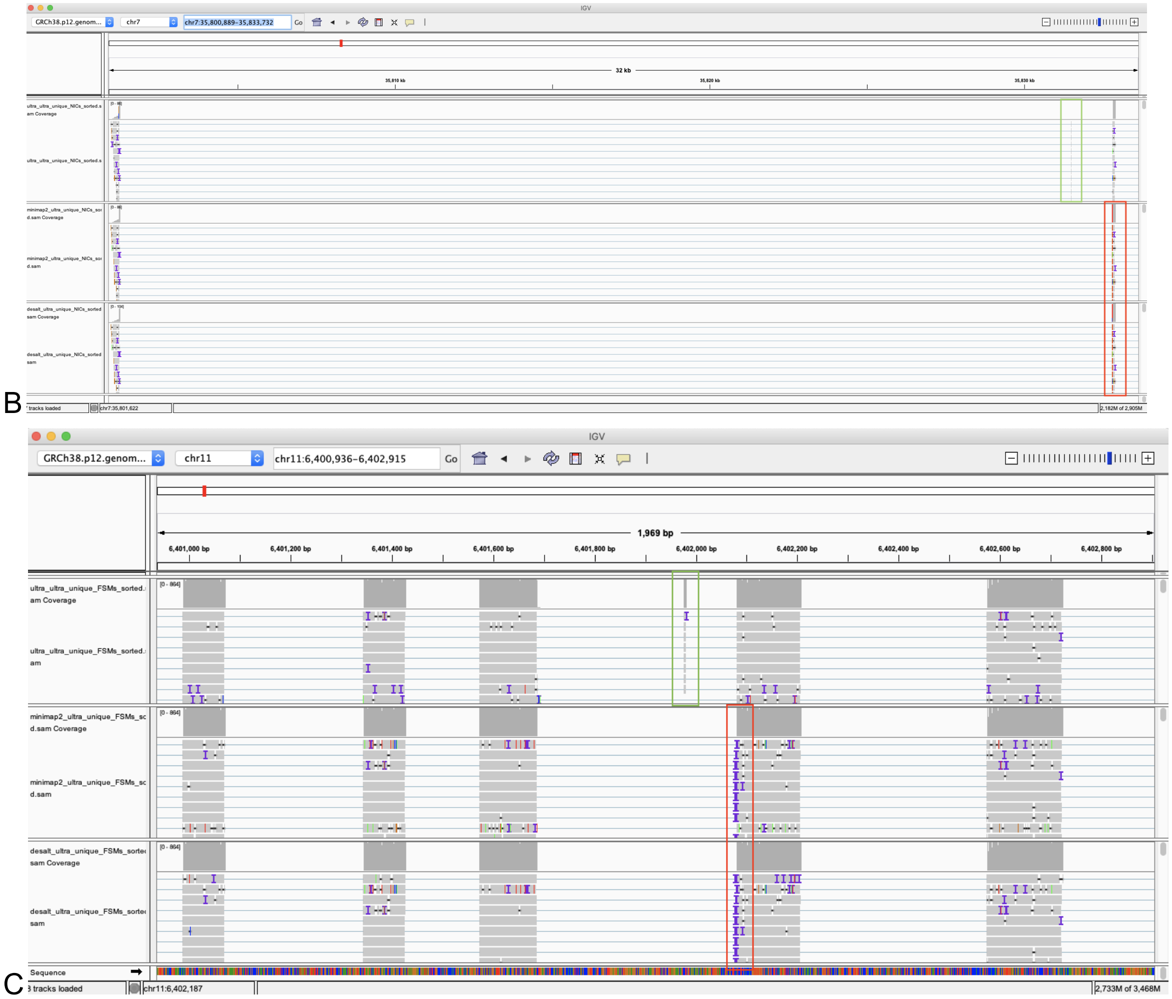

Figure S14. IGV tracks of splice read alignments to unique NIC isoforms found by uLTRA_mm2 (first track) by aligning to small exons (5-6nt), with corresponding aligned reads of minimap2 (second track) and deSALT_GTF (third track). A green rectangle highlights the best fit exon alignments. The red box highlights misalignments around the junctions caused by the unaligned small exons. (A) 126 reads mapping to a NIC transcript (with a 6nt exon) from the MICU1 gene. (B) 81 reads mapping to a NIC (with a 5nt exon) transcript of the SEPTIN7 gene. (C) 25 reads mapping to a NIC transcript (with a 6nt exon) from the APBB1 gene.

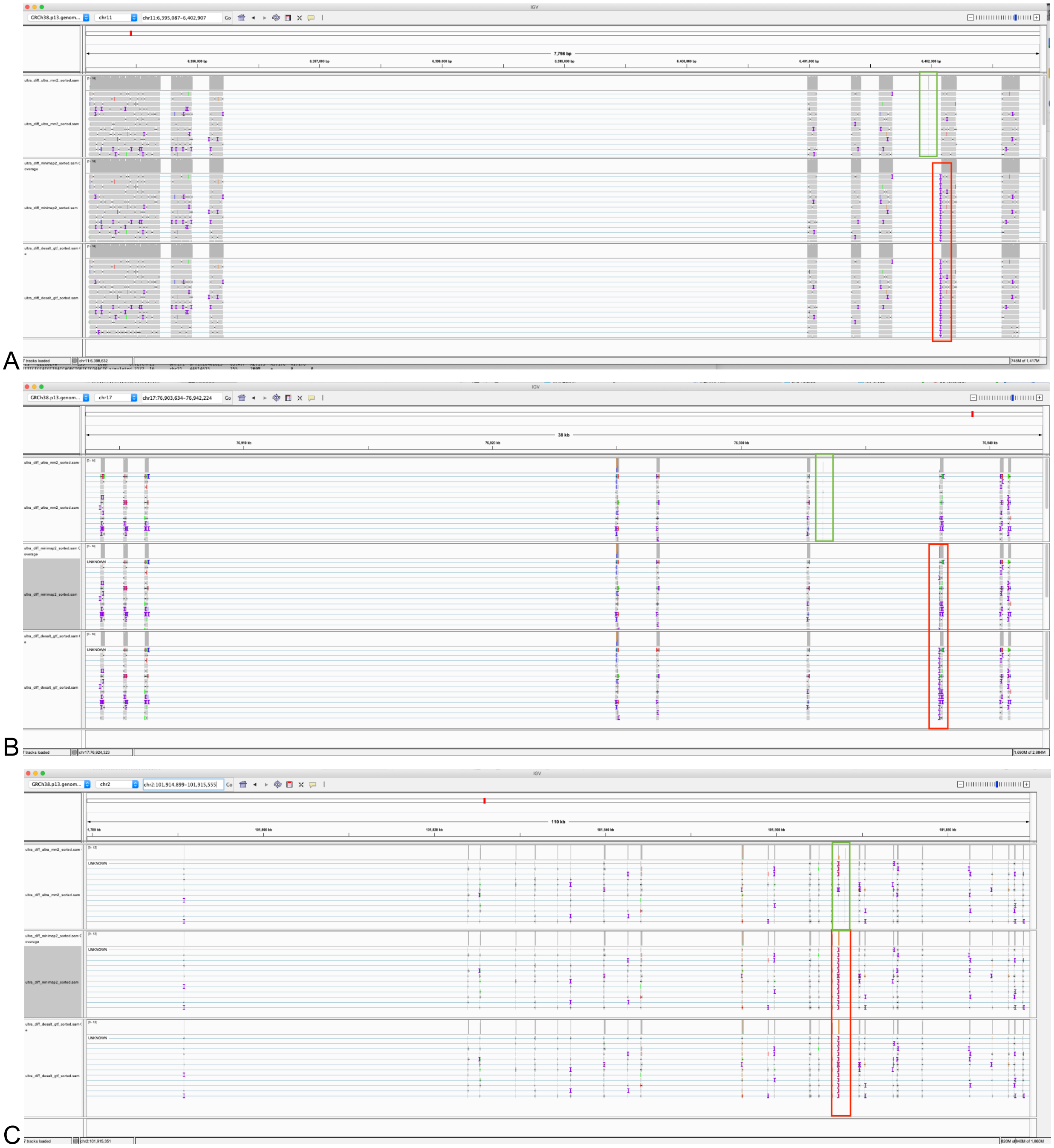

Figure S15. Three examples of small exon misalignments from the set of isoforms in the ALZ dataset with FSM predictions shared by minimap2 and deSALT but not by uLTRA_mm2. uLTRA_mm2 is shown in the first track in each of the figures, while minimap2 is shown in the second track and deSALT_GTF in the third track). The green box highlights the best fit exon alignment. The red box highlights misalignments around the junctions caused by the unaligned small exons.

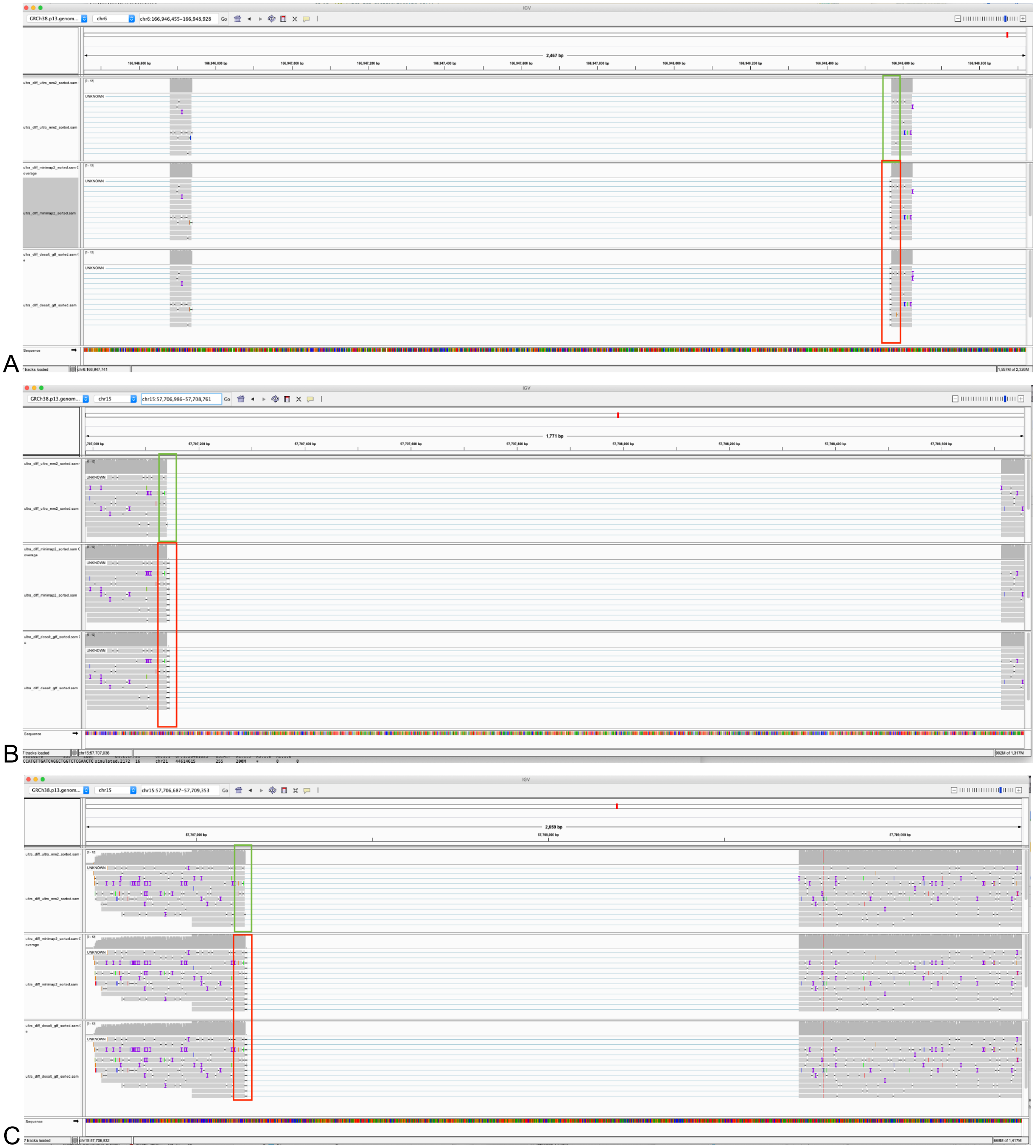

Figure S16. Three examples of over-fitting splice read alignments to canonical splice sites from the set of isoforms in the ALZ dataset with FSM predictions shared by minimap2 and deSALT but not by uLTRA_mm2. uLTRA_mm2 is shown in the first track in each of the figures, while minimap2 is shown in the second track and deSALT_GTF in the third track). The green box highlights the best fit alignment. The red box highlights deletions caused by over-fitting.

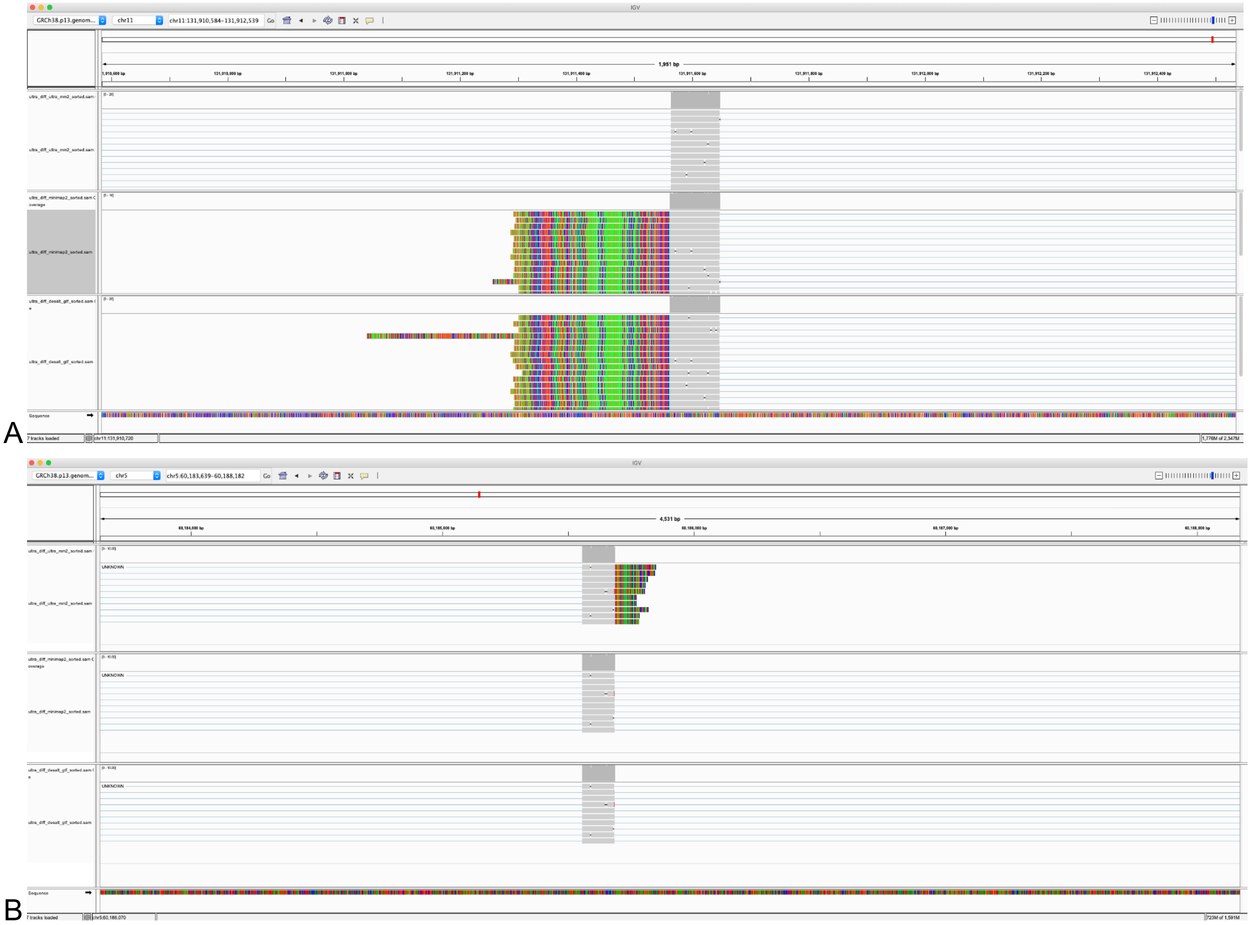

Figure S17. Two examples of the inability to find correct alignment over large introns. (A) uLTRA_mm2 correctly aligns over an intron of size 540,675nt on chromosome 5. (B) uLTRA_mm2 incorrectly softclipps a read with a 302,254nt intron on chromosome 11 that are correctly found by minimap2 and deSALT_GTF.

Supplementary Tables

Dataset	uLTRA	uLTRA_mm2	minimap2	minimap2_GTF	deSALT	deSALT_GTF
ENS	51m	51m	13m	14m	6m	6m
SIM_ANN	1h 21m	1h 45m	45m	47m	23m	23m

SIM_NIC	1h 31m	2h 41m	1h 17m	1h 19m	30m	32m
SIRV	17m	19m	4m	4m	2m	2m
ALZ	4h 49m	5h 42m	2h 36m	2h 48m	2h 42m	2h 41m
DROS	43m	42m	6m	7m	8m	8m

Table S1. Runtime of alignment using 19 cores.

Dataset	uLTRA	uLTRA_mm2	minimap2	minimap2_GTF	deSALT	deSALT_GTF
ENS	15Gb	20Gb	17Gb	22Gb	38Gb	38Gb
SIM_ANN	17Gb	21Gb	18Gb	18Gb	38Gb	38Gb
SIM_NIC	19Gb	23Gb	19Gb	19Gb	39Gb	39Gb
SIRV	6Gb	8Gb	3Gb	3Gb	2Gb	2Gb
ALZ	52Gb	64Gb	20Gb	19Gb	50Gb	40Gb
DROS	12Gb	17Gb	7Gb	6Gb	13Gb	13Gb

Table S2. Peak memory usage of alignment using 4 cores.

Dataset	uLTRA	uLTRA_mm2	minimap2	minimap2_GTF	deSALT	deSALT_GTF
ENS	69Gb	70Gb	28Gb	28Gb	38Gb	38Gb
SIM_ANN	70Gb	74Gb	29Gb	30Gb	40Gb	40Gb
SIM_NIC	70Gb	73Gb	32Gb	32Gb	39Gb	39Gb
SIRV	6Gb	8Gb	5Gb	5Gb	3Gb	4Gb
ALZ	90Gb	102Gb	28Gb	30Gb	136Gb	85Gb
DROS	18Gb	23Gb	11Gb	12Gb	32Gb	32Gb

Table S3. Peak memory usage of alignment using 19 cores.

Dataset	uLTRA	minimap2	deSALT
HG38	23m	5m	2h 49m
SIRV	0m	0m	1m
DROS	3m	0m	9m

Table S4. Runtime of indexing.

Dataset	uLTRA	minimap2	deSALT
HG38	7Gb	19Gb	74Gb
SIRV	0Gb	0Gb	2Gb
Drosophila	3Gb	2Gb	6Gb

Table S5. Peak memory usage of indexing.

References

Bagyinszky, Eva, Min Ju Kang, Jungmin Pyun, Vo Van Giau, Seong Soo A. An, and Sangyun Kim. 2019. "Early-Onset Alzheimer's Disease Patient with Prion (PRNP) p.Val180Ile Mutation." *Neuropsychiatric Disease and Treatment*. https://doi.org/10.2147/ndt.s215277.

Boland, Barry, Wai Haung Yu, Olga Corti, Bertrand Mollereau, Alexandre Henriques, Erwan Bezard, Greg M. Pastores, et al. 2018. "Promoting the Clearance of Neurotoxic Proteins in Neurodegenerative Disorders of Ageing." *Nature Reviews. Drug Discovery* 17 (9): 660–88.

Calvo-Rodriguez, Maria, Steven S. Hou, Austin C. Snyder, Elizabeth K. Kharitonova, Alyssa N. Russ, Sudeshna Das, Zhanyun Fan, et al. 2020. "Increased Mitochondrial Calcium Levels Associated with Neuronal Death in a Mouse Model of Alzheimer's Disease." *Nature Communications*. https://doi.org/10.1038/s41467-020-16074-2.

Charbonnier, F., J. P. Périn, G. Roussel, J. L. Nussbaum, and P. M. Alliel. 1997. “[Cloning of testican/SPOCK in man and mouse. Neuromuscular expression perspectives in pathology].” *Comptes rendus des seances de la Societe de biologie et de ses filiales* 191 (1): 127–33.
Daily, Jeff. 2016. “Parasail: SIMD C Library for Global, Semi-Global, and Local Pairwise Sequence Alignments.” *BMC Bioinformatics* 17 (February): 81.
Fernandes, Francisco, and Ana T. Freitas. 2014. “slaMEM: Efficient Retrieval of Maximal Exact Matches Using a Sampled LCP Array.” *Bioinformatics* 30 (4): 464–71.
Geuens, Thomas, Delphine Bouhy, and Vincent Timmerman. 2016. “The hnRNP Family: Insights into Their Role in Health and Disease.” *Human Genetics* 135 (8): 851–67.
Li, Heng. 2018. “Minimap2: Pairwise Alignment for Nucleotide Sequences.” *Bioinformatics* 34 (18): 3094–3100.
Liu, Bo, Yadong Liu, Junyi Li, Hongzhe Guo, Tianyi Zang, and Yadong Wang. 2019. “deSALT: Fast and Accurate Long Transcriptomic Read Alignment with de Bruijn Graph-Based Index.” *Genome Biology* 20 (1): 274.
Li, Yu, Renmin Han, Chongwei Bi, Mo Li, Sheng Wang, and Xin Gao. 2018. “DeepSimulator: A Deep Simulator for Nanopore Sequencing.” *Bioinformatics* 34 (17): 2899–2908.
Robinson, James T., Helga Thorvaldsdóttir, Wendy Winckler, Mitchell Guttman, Eric S. Lander, Gad Getz, and Jill P. Mesirov. 2011. “Integrative Genomics Viewer.” *Nature Biotechnology* 29 (1): 24–26.
Sahlin, Kristoffer, and Paul Medvedev. 2021. “Error Correction Enables Use of Oxford Nanopore Technology for Reference-Free Transcriptome Analysis.” *Nature Communications*. https://doi.org/10.1038/s41467-020-20340-8.
“SNaReSim: Synthetic Nanopore Read Simulator - IEEE Conference Publication.” n.d. Accessed June 21, 2019. https://ieeexplore.ieee.org/document/8031171.
Šošic, Martin, and Mile Šikic. 2017. “Edlib: A C/C ++ Library for Fast, Exact Sequence Alignment Using Edit Distance.” *Bioinformatics* 33 (9): 1394–95.
Stöcker, Bianca K., Johannes Köster, and Sven Rahmann. 2016. “SimLoRD: Simulation of Long Read Data.” *Bioinformatics*. https://doi.org/10.1093/bioinformatics/btw286.
Tanahashi, Hiroshi, and Takeshi Tabira. 1999. “Molecular Cloning of Human Fe65L2 and Its Interaction with the Alzheimer’s β-Amyloid Precursor Protein.” *Neuroscience Letters*. https://doi.org/10.1016/s0304-3940(98)00995-1.
Tardaguila, Manuel, Lorena de la Fuente, Cristina Marti, Cécile Pereira, Francisco Jose Pardo-Palacios, Hector Del Risco, Marc Ferrell, et al. 2018. “SQANTI: Extensive Characterization of Long-Read Transcript Sequences for Quality Control in Full-Length Transcriptome Identification and Quantification.” *Genome Research*, February. https://doi.org/10.1101/gr.222976.117.
Tian, Yuan, Jerry C. Chang, Emily Y. Fan, Marc Flajolet, and Paul Greengard. 2013. “Adaptor Complex AP2/PICALM, through Interaction with LC3, Targets Alzheimer’s APP-CTF for Terminal Degradation via Autophagy.” *Proceedings of the National Academy of Sciences of the United States of America* 110 (42): 17071–76.
Wang, Xinlu, Fei Fei, Jie Qu, Chunyuan Li, Yuwei Li, and Shiwu Zhang. 2018. “The Role of Septin 7 in Physiology and Pathological Disease: A Systematic Review of Current Status.” *Journal of Cellular and Molecular Medicine* 22 (7): 3298–3307.
Yang, Chen, Justin Chu, René L. Warren, and Inanç Birol. 2017. “NanoSim: Nanopore Sequence Read Simulator Based on Statistical Characterization.” *GigaScience* 6 (4): 1–6.

Faucon, P. C., Balachandran, P., and Crook, S. "SNaReSim: Synthetic Nanopore Read Simulator," *2017 IEEE International Conference on Healthcare Informatics (ICHI)*, Park City, UT, 2017, pp. 338-344, doi: 10.1109/ICHI.2017.98.
